# Nanofiltration Membranes from Poly(sodium-p-styrenesulfonate)/Polyethylenimine Polyelectrolyte Complex Modified with Carbon Nanoparticles for Enhanced Water Treatment

**DOI:** 10.3390/polym17101306

**Published:** 2025-05-10

**Authors:** Mariia Dmitrenko, Olga Mikhailovskaya, Roman Dubovenko, Anton Mazur, Anna Kuzminova, Igor Prikhodko, Konstantin Semenov, Rongxin Su, Anastasia Penkova

**Affiliations:** 1 St. Petersburg State University, 7/9 Universitetskaya nab., St. Petersburg 199034, Russia; st113220@student.spbu.ru (O.M.); r.dubovenko@spbu.ru (R.D.); a.mazur@spbu.ru (A.M.); a.kuzminova@spbu.ru (A.K.); i.prikhodko@spbu.ru (I.P.); 2 Pavlov First Saint Petersburg State Medical University, L’va Tolstogo ulitsa 6–8, St. Petersburg 197022, Russia; semenov1986@yandex.ru; 3 State Key Laboratory of Chemical Engineering, School of Chemical Engineering and Technology, Tianjin University, Tianjin 300072, China; surx@tju.edu.cn

**Keywords:** polystyrene sulfonate, polyethyleneimine, polyelectrolyte complex, carbon nanoparticles, nanofiltration, water treatment

## Abstract

Industrial wastewater poses a significant environmental challenge due to its harmful effects. The development of sustainable membrane processes for water treatment and the environmentally friendly production of polymer membranes is one of the major challenges of our time. An alternative approach is to prepare polyelectrolyte complex (PEC) membranes using the aqueous phase separation (APS) method without the use of toxic solvents. In this work, PEC nanofiltration membranes of poly(sodium-p-styrenesulfonate) (PSS)/polyethylenimine (PEI) modified with carbon nanoparticles (graphene oxide, polyhydroxylated fullerene (HF), multi-walled carbon nanotubes) were developed for enhanced water treatment from anionic food dyes and heavy metal ions. The effect of varying the PSS/PEI monomer ratio, carbon nanoparticles, the content of the optimal HF modifier, and the cross-linking agent on the membrane properties was studied in detail. The changes in the structure and physicochemical properties of the PEC-based membranes were investigated using spectroscopic, microscopic, thermogravimetric analysis methods, and contact angle measurements. The PSS and PEI interactions during PEC formation and the effect of PEI protonation on membrane properties were investigated using computational methods. The optimal cross-linked PEC/HF(1%) (1:1.75 PSS/PEI) membrane had more than 2 times higher permeability compared to the pristine PEC membrane, with dye and heavy metal ion rejection of 99.99 and >97%, respectively.

## 1. Introduction

Water is undoubtedly one of the most essential components of human life and is crucial for the operation of any industrial enterprise [1,2]. However, effluents from various industrial activities pose a significant threat to ecological stability and human health due to their toxic and polluting properties [3]. The increasing contamination of industrial waters with heavy metal ions and dyes has become a global concern. Consequently, wastewater treatment has become an urgent priority that requires effective solutions.

In the context of sustainable development, there is considerable focus on the use of membrane processes and sustainable membrane development to address environmental challenges. Among these, nanofiltration (NF) stands out as a promising and effective method for separating heavy metal ions and dyes. NF is environmentally friendly, easy to manage and automate, and energy efficient, operating at lower pressures than reverse osmosis [4]. However, most commercial NF membranes struggle to retain heavy metal cations and are less effective against high levels of dye contamination, which can reduce their permeability [5,6]. A promising avenue for improvement is the development of polyelectrolyte complex (PEC) membranes. This involves mixing oppositely charged polyelectrolytes using a sustainable preparation method that avoids toxic organic solvents, specifically the aqueous phase separation (APS) approach with salinity or pH changes [7]. In this work, NF PEC membranes based on the well-known poly(sodium-p-styrenesulfonate) (PSS)/polyethylenimine (PEI) were developed with improved properties through modification by the introduction of carbon nanoparticles (CPs) such as graphene oxide (GO), polyhydroxylated fullerene (HF), and multi-walled carbon nanotubes (MWCNTs). They are of interest as modifiers because of their mechanical strength, chemical stability, and ability to interact with the polymer matrix through functional (oxygen-containing) groups, forming channels for the transport of components. Due to its peculiar structure, the incorporation of this material into the membrane also imparts a negative charge, which enhances the rejection of negatively charged molecules (most natural contaminants are negatively charged [7]) and significantly modifies the structure and properties of the membranes, thereby improving the transport parameters. Despite their potential as modifiers, there is limited research on PEC membranes modified with GO [8,9], HF [10], and MWCNT [11], and there is no information on the modification of PEC from PSS/PEI with these CP.

The membranes based on PEC from PSS/PEI have been developed for filtration in [12,13,14,15,16,17]. PEC from PSS/PEI is more often used for the development of polyelectrolyte multilayer (PEM) membranes for NF of micropollutants, humic acid (HA), monovalent and divalent ions (MgCl_2_ and Na_2_SO_4_), and dye solutions [12,13,14,15]. PEC (PSS/PEI) membranes have been prepared using the pH shift-induced APS method [16,17]. The conditions for membrane preparation, such as changing the molecular weight of PEI, the concentration and pH of the buffer solution in a coagulation bath, and the effect of the cross-linking agent glutaraldehyde (GA), have been studied in detail [16]. The PEC membranes prepared with PEI (25 kDa) at pH 4 had a water permeability of 4 L/(m^2^·h·bar) and a rejection of 45, 87, and 64% for negatively charged molecules sulfamethoxazole, naproxen, bezafibrate, respectively. The performance of PEC membranes prepared from PSS and branched PEI (25 kDa) by the pH shift-induced aqueous phase separation (APS) method was tested in the filtration of salt (MgCl_2_, MgSO_4_, Na_2_SO_4_, NaCl), bovine serum albumin (BSA), and HA solutions [17]. This study investigates the effects of the casting solution temperature and the mixing monomer ratio of PSS/PEI (from 1:1 to 1:1.8) on membrane properties. Optimal mixing ratios of 1:1.70 resulted in NF membranes with a water permeability of approximately 8 LMH/bar. To the best of our knowledge, NF membranes made from PEC of PSS/PEI have not been tested for water purification from dyes and heavy metal ions. Low-molecular-weight PEI has also not been tested, as the molecular weight of polyelectrolyte significantly affects the structure of the resulting PEC.

Thus, the novelty and advantages of this work over previously published works are that, for the first time, (1) low-molecular-weight PEI (10 kDa) is used to prepare PEC from PSS/PEI using the APS method, which contributes to improved permeability, and (2) PSS/PEI membranes modified with CP such as HF, GO, and MWCNT are developed to improve NF water treatment targeting dyes and heavy metal ions. The effect of the PSS/PEI monomer ratio, the type of CP, and the content of the optimal modifier (HF) in the PEC matrix on membrane performance and characteristics was investigated. A number of analytical methods were used to characterize the resulting PEC-based membranes, including NF tests with aqueous solutions of anionic food dyes and heavy metal ions, FTIR and NMR spectroscopies, scanning electron microscopy (SEM), atomic force microscopy (AFM), thermogravimetric analysis (TGA), and contact angle measurements. In addition, theoretical analyses using computational techniques were carried out to elucidate membrane performance and component interactions. This comprehensive approach underlines the importance of the findings for the advancement of water treatment technologies.

## 2. Materials and Methods

### 2.1. Materials

The PEC membranes were prepared using poly(sodium-p-styrenesulfonate) (PSS, 1000 kDa) from Shanghai Macklin Biochemical Technology Co. (Shanghai, China) and branched polyethylenimine (PEI, 10 kDa) from Macklin Co. (Shanghai, China). To improve the performance of the PEC-based membranes, various carbon nanoparticles (CPs) were incorporated as modifiers. These include graphene oxide (GO, synthesized from graphite via oxidation according to modified Hummers and Offeman’s method), polyhydroxylated fullerene (fullerenol, HF, C_60_(OH)_22–24_), and multi-walled carbon nanotubes (MWCNT with specific surface area of 276 m^2^/g) obtained from Fullerene Technologies located in St. Petersburg (Russia). Detailed characterization of GO, HF, and MWCNT is presented in previous works [18,19,20]. Sodium acetate (CH_3_COONa) with 99% purity from LenReactive (St. Petersburg, Russia) and acetic acid (AcOH) from Vekton (St. Petersburg, Russia) were used to prepare a buffer solution. Glutaraldehyde (GA) from LLC TD Gala-Trade (St. Petersburg, Russia) was used as a cross-linking agent.

### 2.2. Membrane Fabrication

**Figure 1:**
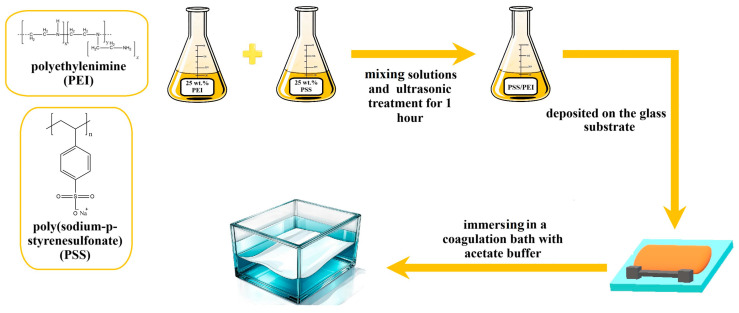
Schematic illustration of the PEC-based membrane preparation.

The preparation of PEC-based membranes was carried out according to the pH shift induced and organic solvent-free aqueous phase separation (APS) process. Aqueous solutions of 25 wt.% PSS and PEI were prepared, mixed to obtain different monomer ratios of PSS/PEI (1:1.5, 1:1.75, and 1:2) to prepare 25 wt.% casting solutions and subjected to ultrasonic treatment for 1 h. The molecular weights of the monomers (PSS~206.19 Da and PEI~43.04 Da per unit of ethyleneimine) were used to calculate the molar mixing ratio. For example, to prepare 20 g of 25 wt.% PSS/PEI (1:1.75), 14.64 g of 25 wt.% PSS solution and 5.36 g of 25 wt.% PEI solution must be mixed. The resulting solution was deposited onto the glass substrate using a 200 µm slit casting blade and then immersed in a coagulation bath containing acetate buffer (0.5 M, pH 4.0) at room temperature. In this bath, the PEC solidified to form a porous membrane structure (Figure 1). The resulting PEC-based membranes were removed from the glass substrate and placed in another bath containing distilled water for at least 24 h.

The modification of PEC-based membranes with a PSS/PEI ratio of 1:1.75 was carried out by introducing 1 wt.% of GO, HF, and MWCNT nanoparticles with respect to the total PEC mass. The HF and GO modifiers were added to the PEC solution as an aqueous dispersion at a concentration of 20 g/L. MWCNTs were introduced via mechanical grinding with PSS powder in the first step of solution preparation [21]. Mechanical grinding is most commonly used to disperse agglomerated MWCNT [22,23] and does not have a significant effect on the particles [24]. The introduction of pristine MWCNT particles into a concentrated 25 wt.% solution of high-molecular-weight PSS (1000 kDa) and treatment with ultrasound alone did not result in uniform dispersion of the modifier, resulting in the formation of PEC membranes with defects. In addition, all solutions of PSS/PEI/CP composites obtained were subjected to ultrasonic treatment for 1 h prior to membrane formation by the APS method.

Cross-linking of PEC-based membranes was performed as follows: 0.01 wt.% GA was added to a coagulation bath of acetate buffer [17]. GA reacted with the primary amine groups of PEI, forming an imine bond through the Schiff base reaction [16,25]. The PEC-based membrane precipitated in the coagulation bath with GA was kept for 5 h, then removed and stored in water for further characterization and testing. Table 1 summarizes the names of the PEC-based membranes obtained and their compositions.

### 2.3. Nanofiltration Performance

**Figure 2:**
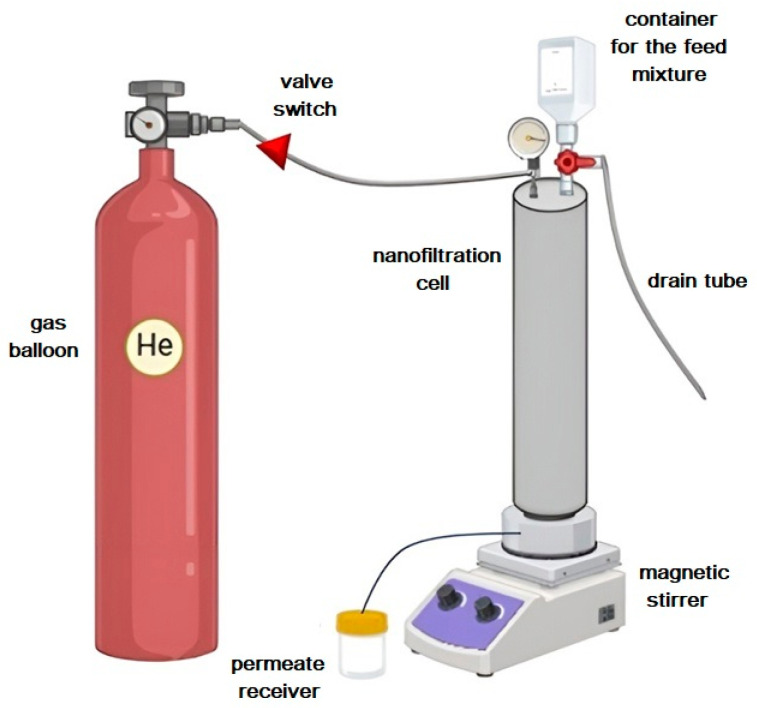
Nanofiltration setup scheme.

Laboratory nanofiltration tests were carried out using a dead-end filtration cell with an effective area of 0.2 × 10⁻^2^ m^2^. The experiments were carried out under ambient temperature conditions and an applied transmembrane pressure of up to 50 atm. To ensure a uniform concentration distribution and to avoid concentration polarization effects, continuous agitation was applied to the 500 mL feed solution. A visual representation of the experimental setup is shown in Figure 2. Extensive testing was carried out for each membrane sample over a minimum of 7 days. To ensure consistency of membrane properties, water permeation tests were performed both before and after the contaminate solution filtration. After one week of continuous monitoring, the collected data were averaged. The average accuracy of the membrane transport parameters was ±5% for permeability and ±0.5% for rejection coefficient.

The permeability of the membrane was determined using Equation (1) [26]:

(1) $$L=\frac{m}{A\cdot t\cdot ∆P},$$

where *m* represents the mass of the permeate (kg), *t* is the duration of permeate collection (h), *A* is the effective surface area of the membrane (m^2^), and ∆*P* denotes the transmembrane pressure (atm).

The rejection coefficient was calculated using Equation (2):

(2) $$R=\left(1-\frac{C_{perm}}{C_{feed}}\right)\cdot 100\%,$$

where *C_perm_* and *C_feed_* indicate the concentrations of the components in the permeate and the feed, respectively.

The flux recovery ratio (FRR) was calculated using Equation (3):

(3) $$FRR=\left(\frac{L}{L_{water}}\right)\cdot 100\%$$

where *L* is the pure water permeability after the feed permeation through the membrane, and *L_water_* is the initial pure water permeability.

The primary application of the developed PEC-based membranes was evaluated through nanofiltration tests for the removal of dye contaminants from aqueous solutions. The study used a range of food-grade anionic dyes to assess membrane performance. The test dyes included Sunset Yellow (SY), Congo Red (CR), and Alphazurine (AZ), all prepared as aqueous solutions at a standardized concentration of 10 mg/L [27]. Detailed information on these dyes is presented in Table 2. To quantify the dye concentration in both the feed solution and the permeate, a PE-5400UV spectrophotometer manufactured by EKROSKHIM Co. (St. Petersburg, Russia) was employed. Spectral analysis was performed at specific wavelengths corresponding to the maximum absorbance values listed in Table 2 to ensure accurate and reliable measurements for each dye.

The cross-linked PEC and PEC/HF membranes were rigorously tested to evaluate their efficacy in nanofiltration of heavy metal ion solutions. The test solutions contained Cu(NO_3_)_2_, Pb(NO_3_)_2_, and Cd(NO_3_)_2_ at a concentration of 50 mg/L each [3]. The filtration cell was thoroughly cleaned with a 5 g/L aqueous Trilon B solution to ensure consistent performance and to prevent cross-contamination. Stripping voltammetry was used to accurately determine the concentration of metal ions in both feed and permeate using a TA-4 voltammetric analyzer from Tomanalit (Tomsk, Russia). The electrochemical setup consisted of silver chloride electrodes acting as both reference and auxiliary electrodes, supplemented by a mercury film electrode acting as the working electrode. Each membrane was continuously tested for a minimum of seven days to collect comprehensive performance data. The results presented are based on averaged measurements taken over this period, ensuring reliable and reproducible results.

### 2.4. Membrane Characterization

The structure of the PEC-based membranes was investigated using spectroscopic methods—Fourier transform infrared (FTIR) and nuclear magnetic resonance (NMR) spectroscopy. The PEC-based membranes were analyzed using a Shimadzu IRAffinity-1S spectrometer from Kyoto (Japan), together with an attenuated total reflectance (ATR) instrument from PIKE Technologies (Madison, WI, USA). The study was carried out within a frequency range of 450 to 4000 cm^−1^ while maintaining a constant temperature of 25 °C. The membranes were analyzed using a Bruker Avance III 400 WB spectrometer manufactured by Bruker in Billericay (MA, USA). This spectrometer operates at a magnetic field strength of 9.4 T and is equipped with a 4 mm CP/MAS probe. For all experiments, the Magic Angle Spinning (MAS) frequency was set to 10 kHz. Analysis of the ^13^C nuclei was performed at a Larmor frequency of 100.64 MHz using tetramethylsilane (TMS) as an external reference standard.

The cross-sectional and surface morphology was studied using scanning electron microscopy (SEM) and atomic force microscopy (AFM). The structure of the PEC-based membranes, both at the surface and in cross-section, was analyzed using a Zeiss AURIGA laser system from Carl Zeiss SMT (Oberkochen, Germany). This laser operated at a voltage of 30 kV and a current of 2 pA. For the cross-sectional samples, the membranes were immersed in liquid nitrogen before being carefully broken. The surface of the membranes was thoroughly examined using an NT-MDT NTegra Maximus system from NT-MDT Spectrum Instruments (Moscow, Russia). Tapping mode was used to obtain accurate and reliable results. Measurements were made using standard silicon cantilevers with a stiffness of 15 N/m.

The thermochemical properties of PEC-based membranes were investigated using a TG 209 F1 Libra thermobalance supplied by Netzsch (Selb, Germany). The evaluation was carried out in an argon atmosphere at a heating rate of 10 °C/min, using samples weighing between 2 and 4 mg. The water contact angle for PEC-based membranes was measured using the sessile drop method using an LK-1 goniometer (OOO “NPK Open Science”, Krasnogorsk, Russia). The contact angle values were analyzed using DropShape software (https://drop-shape-analysis.software.informer.com/2.5/). Membranes were evaluated from both sides.

### 2.5. Computational Methods

The Gaussian 16, Revision A.03 [28] software package was utilized for all calculations in this study. Geometries were thoroughly optimized using the B3LYP [29,30,31] level of theory and the standard 6-311++G(d,p) basis set. Subsequently, harmonic vibrational frequency analysis was conducted at the same theoretical level to characterize all geometries. When a saddle point was identified, the mode corresponding to the first imaginary frequency was used to locate the local minimum. All reported structures in this study have been confirmed to be true local minima. It is imperative to note that no symmetry constraints were applied during the course of the calculations. The singlet state was chosen as the ground state. The thermodynamic properties were calculated at 1 atm and 298.150 K. The obtained wave functions were processed using the multifunctional wavefunction analyzer (Multiwfn 3.8 [32], release date: 17 April 2024). The visualization process was executed through the utilization of VMD software (version 1.9.4a53, release date: 29 June 2021) [33].

## 3. Results

This section consists of four main subsections. Section 3.1 is dedicated to the optimisation of PEC-based membranes for enhanced nanofiltration by variation of the PSS/PEI ratio (Section 3.1.1), modification of the PEC membrane with an optimal PSS/PEI ratio with CP (Section 3.1.2), and cross-linking of the PEC/HF membrane with GA (Section 3.1.3), which demonstrated the improved properties. Section 3.2 describes the study of changes in the structural and physicochemical properties of the membranes using different analytical methods during the modification process. Quantum chemical studies are presented in Section 3.3 to observe interactions between polymers and separated components. To demonstrate the potential of the developed membranes, they were compared with PEC-based membranes published in the literature (Section 3.4).

### 3.1. Transport Characteristics in Nanofiltration

#### 3.1.1. Variation in PSS/PEI Ratio in Membrane Composition

**Figure 3:**
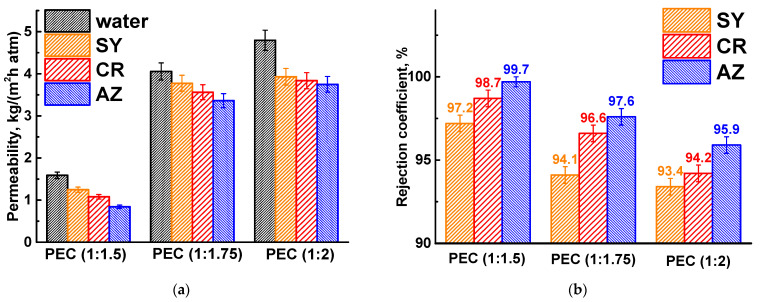
(**a**) Permeability and (**b**) rejection coefficient of dyes for PEC-based membranes prepared using different PSS/PEI ratios.

The composition of the PEC membranes was varied by changing the ratio of PSS/PEI (1:1.5, 1:1.75, and 1:2) in the casting polymer solution during membrane formation. These ratios were chosen on the basis of previous works [16,17]. It should be noted that in this work, a lower-molecular-weight branched PEI (10 kDa) was initially used without cross-linking, which, in turn, significantly affected the transport properties of the resulting PEC membranes. The transport properties of the developed PEC membranes with different PSS/PEI ratios were investigated in the nanofiltration of dye solutions (Figure 3).

The permeability and rejection characteristics of PEC-based membranes are influenced by the molecular weight of the dyes [34]. As the dye molecular weight increases, permeability tends to decrease due to fouling effects, while dye rejection improves. Several mechanisms contribute to dye retention, including variations in diffusion and solubility, the molecular sieve effect, and the Donnan effect [35]. The separation of dye molecules is primarily controlled by their molecular weight (sieve effect) and electrostatic interactions (charge). Consequently, as the molecular weight of the dyes increases (Table 2), there is a corresponding increase in the rejection coefficient and a decrease in the permeability of the PEC membranes. With increasing PEI in the PEC composition, the permeability of the membrane increased, and their dye rejection capacity decreased. This may be due to a more favorable interaction of cationic PEI with water (confirmed by computational methods in Section 3.3), the formation of a more open porous structure, a larger pore surface area for component penetration, as well as an increase in positive surface charge due to PEI (confirmed by computational methods in Section 3.3) [16,17]. These changes contributed to increased water penetration and attracted more negatively charged dyes, which passed through the membrane with the water. Based on the performance data, the PEC membrane prepared from the casting solution of PSS/PEI (1:1.75) was selected as optimal for further modification with CP due to the optimal ratio of permeability and rejection coefficients: improved permeability by more than 2.6 times with a slight decrease in dye retention (less than 3.1%) compared to the PEC (1:1.5) membrane. It should also be noted that membranes prepared at PSS/PEI ratios of 1:2 formed patterned surfaces due to excess PEI [17].

#### 3.1.2. Modification of PEC with Carbon Nanoparticles

**Figure 4:**
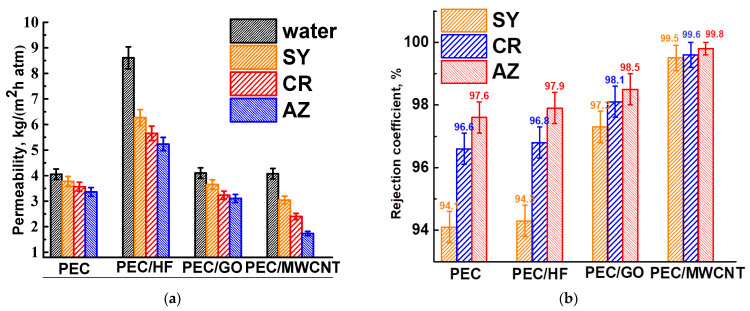
(**a**) Permeability and (**b**) rejection coefficient of dyes for PEC membranes modified with CP (HF, GO, and MWCNT).

To investigate the effect of modifying the PEC membrane with optimal properties, 1 wt.% of CP, such as HF, GO, and MWCNT, was incorporated into the PEC matrix. These modified membranes were then tested for nanofiltration of anionic dye solutions (Figure 4). The parameters of the PEC membrane are also shown in Figure 4 for comparison.

The introduction of CP into the matrix of the PEC membrane resulted in an increase in permeability and dye rejection coefficients. The improved retention of the modified membranes can be attributed to the anionic repulsion between CP and dyes within the membranes. Incorporation of CP with a high concentration of oxygen-containing functional groups (particularly hydroxyl and carboxyl) increases the negative charge on the membrane surface, resulting in greater electrostatic repulsion [34]. The PEC/GO and PEC/MWCNT membranes have close permeability values and are significantly lower than the PEC/HF membrane due to the formation of a denser upper layer for these membranes (confirmed by SEM data below), which impedes transport across the membranes. However, MWCNT and GO, when used as modifiers, can create selective channels for water due to their hydrophilic properties (confirmed by contact angle data below), thereby facilitating efficient permeability [36]. It is worth noting that the permeability of dye solutions for the PEC/MWCNT membrane decreases significantly compared to the PEC/GO membrane, but its rejection coefficients also increase. This may be due to the formed surface structure of the PEC/MWCNT membrane, on which small pores have formed (confirmed by SEM data below), providing high selectivity but also strong contamination with dyes, leading to a decrease in membrane permeability [37]. The PEC/HF membrane has the highest permeability of all modified membranes and slightly improved dye rejection coefficients compared to the pristine PEC membrane. These changes in performance were due to the formation of spongy-like inner structure of the membrane (confirmed by SEM data below), which provided a larger effective contact and transport area, and the most hydrophilic surface (confirmed by contact angle data below) due to the largest amount of functional HF groups, which facilitated water penetration [10]. Thus, HF was chosen as the optimal modifier for the PEC membrane among all CP.

To study the effect of this nanofiller, PEC-based membranes modified with different HF contents (1, 3, and 5 wt.%) were also tested (Figure S1 in the Supplementary Materials). It was shown that increasing the HF concentration in the membrane led to a decrease in permeability and an increase in dye rejection coefficients. This can be attributed to the ability of HF to act as a structuring and cross-linking agent for polymer chains [38], particularly for PEI. This interaction forms a robust hydrogen bonding system (confirmed by FTIR data below) and contributes to a more amorphous state of the matrix (confirmed by NMR data below) [38] while also increasing the negative charge on the surface. Based on the transport data obtained, the optimal membrane was identified as PEC/HF (1 wt.%), which resulted in a twofold increase in permeability while maintaining rejection levels comparable to those of the pristine PEC membrane.

#### 3.1.3. Cross-Linking of PEC and PEC/HF Membranes

**Figure 5:**
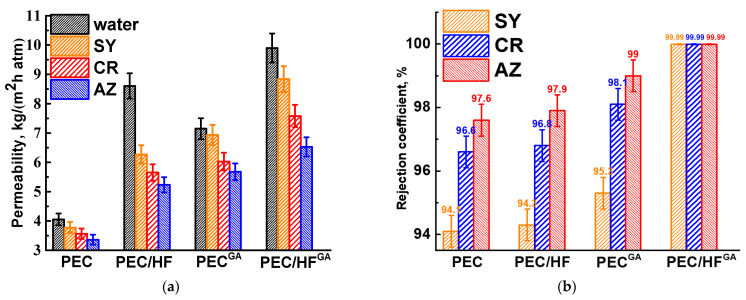
(**a**) Permeability and (**b**) rejection coefficient of dyes for untreated and cross-linked membranes based on PEC and PEC/HF (1%) composite.

PEC and PEC/HF membranes were cross-linked with GA to increase mechanical strength and long-term stability for potential industrial applications [17]. A comparison of untreated and cross-linked membranes in the nanofiltration of dye solutions is shown in Figure 5.

Cross-linking of the membranes with GA resulted in increased permeability and dye retention. This can be explained by the formation of a more open internal porous structure with a thinner upper selective layer after cross-linking (confirmed by SEM data below) and surface hydrophilization (confirmed by contact angle data below). The cross-linked modified PEC/HF^GA^ membrane had more than 2 times higher permeability and high dye rejection (99.99 wt.%) compared to the unmodified PEC membrane. These alterations were caused by changes in the morphology, surface charge, and structure during the HF modification and cross-linking process. The fouling of this PEC/HF^GA^ membrane was evaluated in terms of the FRR parameter calculated from nanofiltration experiments performed with each dye solution over a 24 h period (alternating water and dye solution). It was shown that the FRR values were 94%, 90%, and 86% after the nanofiltration experiments with SY, CR, and AZ solutions, respectively. The decrease in the FRR parameters is due to the increase in the molecular weight of the dyes, which contributes to a greater contamination of the membrane.

**Figure 6:**
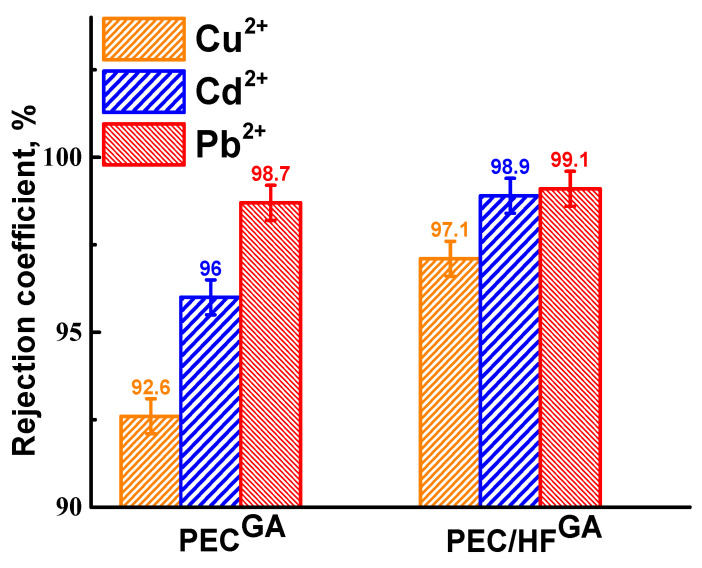
Rejection coefficient of heavy metal ions for cross-linked PEC and PEC/HF membranes.

For potential industrial applications in wastewater treatment, the PEC/HF^GA^ membrane was tested in nanofiltration of aqueous solutions containing heavy metal ions (Figure 6). The pristine PEC membrane was also evaluated for comparison. The permeability of heavy metal ions solutions was not reported separately, as it was consist with the water permeability values previously shown in Figure 5.

The PEC/HF^GA^ membrane showed the highest rejection, achieving retention rates of 97.1% for Cu^2+^, 98.9% for Cd^2+^, and 99.1% for Pb^2+^. In contrast, the PEC membrane exhibited reduced rejection. The metal rejection coefficients correlated with the size of the metal ions [39]. In addition, the rejection of Cu^2+^ was lower for the membranes compared to the other metal ions. This may be due to the formation of complexes between copper and donor atoms [3]. Therefore, the nanofiltration cross-linked PEC/HF^GA^ membrane was developed to improve the performance in the treatment of water contaminated with dyes and heavy metal ions.

### 3.2. Structure and Properties Investigation of PEC Membranes

**Figure 7:**
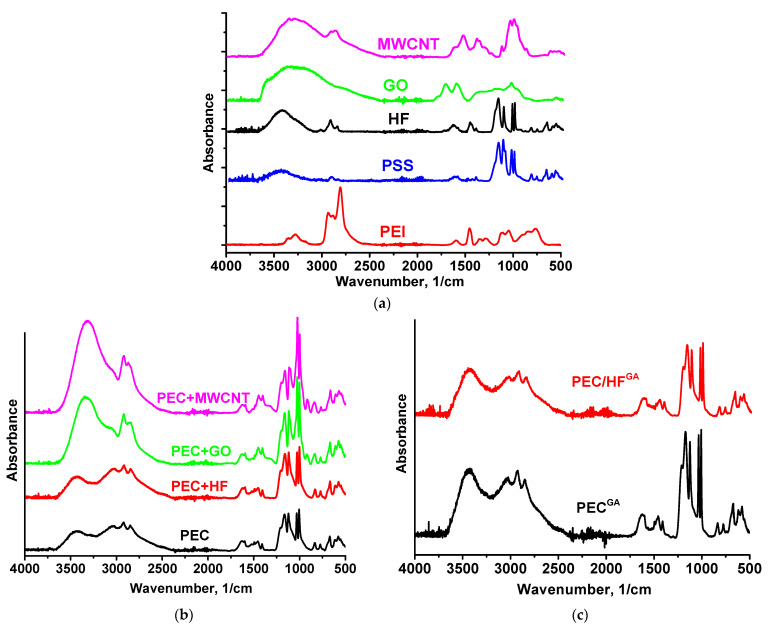
FTIR spectra for (**a**) components (PEI, PSS, HF, GO, and MWCNT), (**b**) untreated PEC and PEC/CP(1%) membranes, and (**c**) cross-linked PEC and PEC/HF(1%) membranes.

The structure of PEC-based membranes was studied using spectroscopic methods (FTIR and NMR). The FTIR spectra of the components (PEI, PSS, HF, GO, and MWCNT) and the membranes obtained are shown in Figure 7.

The vibrational modes in the 700–1700 cm^−1^ range mainly consist of a combination of NH bending and CH_2_ motions [40]: a distinct peak at 1596 cm^−1^ is associated with amines [41,42], and CN stretching and NH bending mixed with CH_2_ scissors are characterized by peaks at 1113 and 1455 cm^−1^. NH stretching, known for its high sensitivity to hydrogen bonding interactions, occurs in the range of 3000 to 3400 cm^−1^ [40]. As the strength of the hydrogen bond increases, these frequencies decrease. The involvement of functional groups in intermolecular interactions (particularly hydrogen bonding) results in a decrease in peak intensity and broadening due to changes in bond lengths and angles [43]. The CH and CH_2_ vibrational peaks are observed in PEI in the region of 2800–2950 cm^−1^. The FTIR spectrum of PSS has main characteristic peaks at 1174, 1039 cm^−1^ (corresponding to the antisymmetric and symmetric vibrations of the SO_3_ group), and 1126 cm^−1^ related to the in-plane benzene ring vibration [17,44]. The main characteristic peaks of HF were located at 3391 (O–H), 1576 (C=C), and 1375 (O–H bending vibration) cm^−1^ [21,45,46]. Main GO peaks at 3373 cm^−1^ (O–H), 1730 cm^−1^ (C=O), 1614 cm^−1^ (C=C), and 1042 cm^−1^ (C–O, epoxy groups) were observed [18,47,48]. The spectrum of MWCNT demonstrated peaks at 1383 cm^−1^ and 1122 cm^−1^ (C=C deformation vibrations), 3351 cm^−1^ (O–H), 2919 cm^−1^ and 2871 cm^−1^ (C–H), and 1650 cm^−1^ (C=C stretching vibrations) [49,50,51,52,53,54].

**Figure 8:**
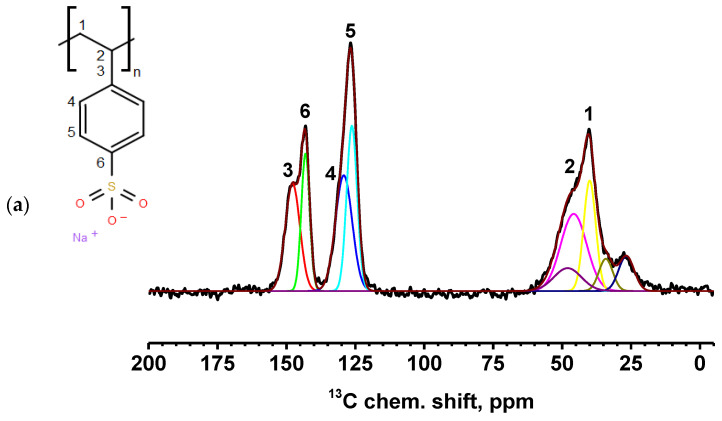

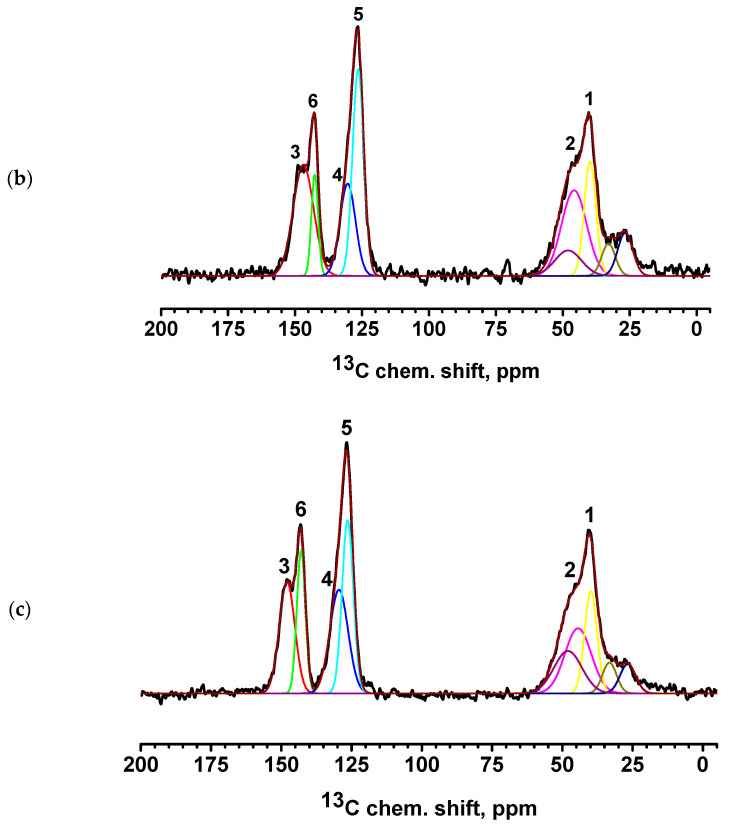
^13^C NMR spectra of PEC membranes with different monomer ratios of PSS/PEI: (**a**) 1:1.5, (**b**) 1:1.75, and (**c**) 1:2. Carbon positions: 1 at 40 ppm, 2 at 45 ppm, 3 at 147 ppm, 4 at 131 ppm, 5 at 126 ppm, and 6 at 142 ppm [56,57].

**Figure 9:**
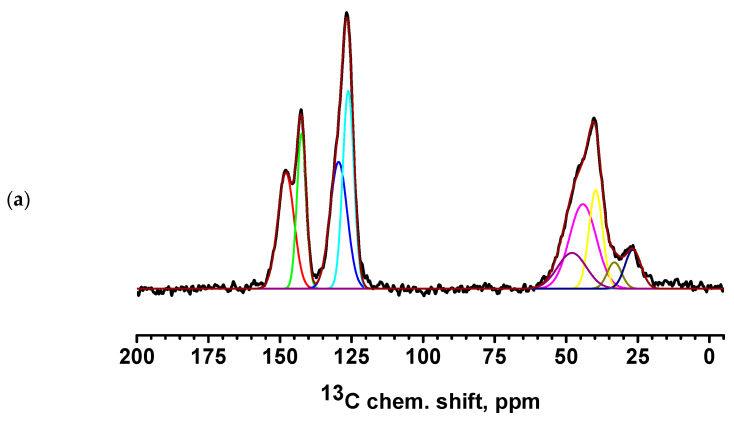

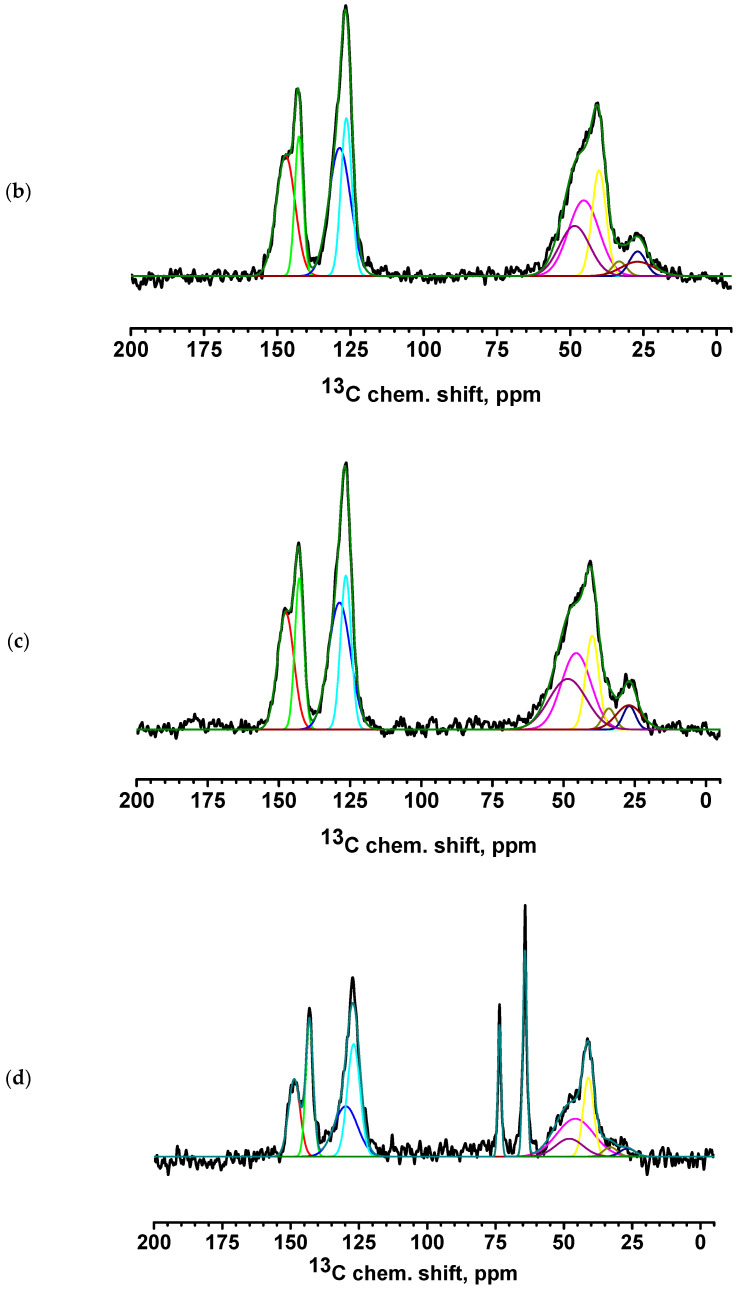

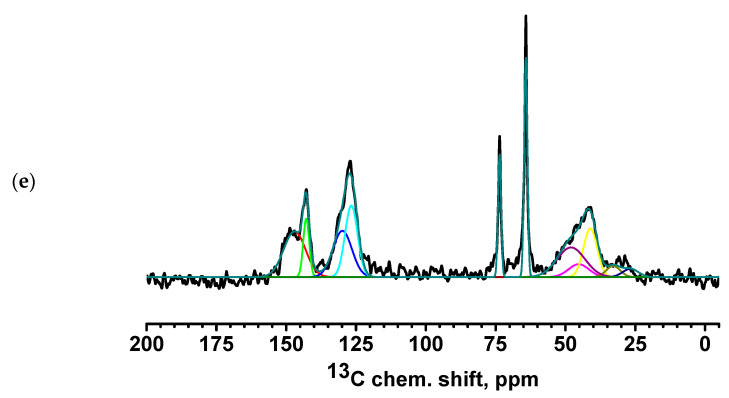
^13^C NMR spectra of PEC-based membranes: (**a**) PEC/HF, (**b**) PEC^GA^, (**c**) PEC/HF^GA^, (**d**) PEC/GO, and (**e**) PEC/MWCNT.

The FTIR spectrum of the PEC membrane reflects the combined spectra of the pristine PEI and PSS polymers. The spectrum showed characteristic peaks at 3432 (NH stretching), 2931 (CH stretching), 1163 (CN stretching), 670 (NH bending), and 1031 and 1005 cm^−1^ (sulfonate moieties) [17]. The introduction of 1 wt.% CP into the PEC membrane resulted in the main peaks of the modifier overlapping with the matrix peaks. For the membranes containing MWCNT and GO, the spectra remained nearly identical to that of pristine PEC, although there was a significant increase in the intensity of the hydroxyl group peak, which shifted from 3434 cm^−1^ to 3312 cm^−1^ and 3350 cm^−1^, respectively, likely due to residual moisture. The following changes were observed for the PEC/HF membrane: an increase in the intensity of the peak at 3434 cm^−1^ and its shift, a shift of the peaks to 3027 and 1627 cm^−1^, and a decrease in the intensity of the peak at ~2000 and 1599 cm^−1^ (primary RNH_3_^+^), indicating the presence of hydrogen bonding between HF and PEC components [21,38]. In addition, it may also be assumed that cationic PEI can form electrostatic interactions with anionic PSS and functional groups of HF [55]. Cross-linking of PEC and PEC/HF membranes with GA resulted in the following changes: a shift and increase in intensity of peaks at 3432 and 3434 cm^−1^, and the broadening of peaks at 1633 and 1628 cm^−1^ due to the formation of a peak at 1660 cm^−1^ (C=N stretching) and a decrease in the primary amine (NH) peak at 1600 cm^−1^. This indicated the formation of imine bonds after the Schiff base reaction between the primary amine of PEI and GA [17,25]. The NMR spectra of PSS, PEI, HF, and PEC-based membranes are shown in Figure S2 in the Supplementary Materials and Figure 8 and Figure 9, respectively.

All spectra for PEC-based membranes with different monomer ratios correspond to the PSS spectra (Figure S2 of Supplementary Materials) [56,57]. When the spectra were decomposed into individual spectral components, a broad component at about 48.5 ppm was added, which was the sum of all signals from liquid PEI (Figure S2 of Supplementary Materials) [58]. It is evident that as the PEI content in the PEC composition increases, the relative intensity of the line corresponding to PEI also increases.

The spectra of the PEC/HF, PEC/GO, and PEC/MWCNT membranes have a slightly broadened line consisting of two components, corresponding to carbon atoms in positions 3 and 6 of PSS. This may indicate the influence of CP molecules on the bonds of PSS and PEI in the direction of amorphization. Two intense new peaks for the PEC/GO and PEC/MWCNT membranes are likely due to the presence of a large amount of physically adsorbed glycerol (which was used to preserve membranes). In the spectrum of the cross-linked PEC^GA^ membrane, a contribution to the spectrum in the region of 26 ppm is observed in the form of a low intensity broad component corresponding to carbon atoms from GA [59]. At the same time, the spectral component corresponding to carbon atoms at position 6 is significantly narrowed. This may indicate an increase in PSS/PEI bonds. In the cross-linked PEC/HF^GA^ membrane, the line from GA is narrowed compared to the PEC^GA^ membrane. This may indicate an increase in the structural homogeneity of the film.

**Figure 10:**
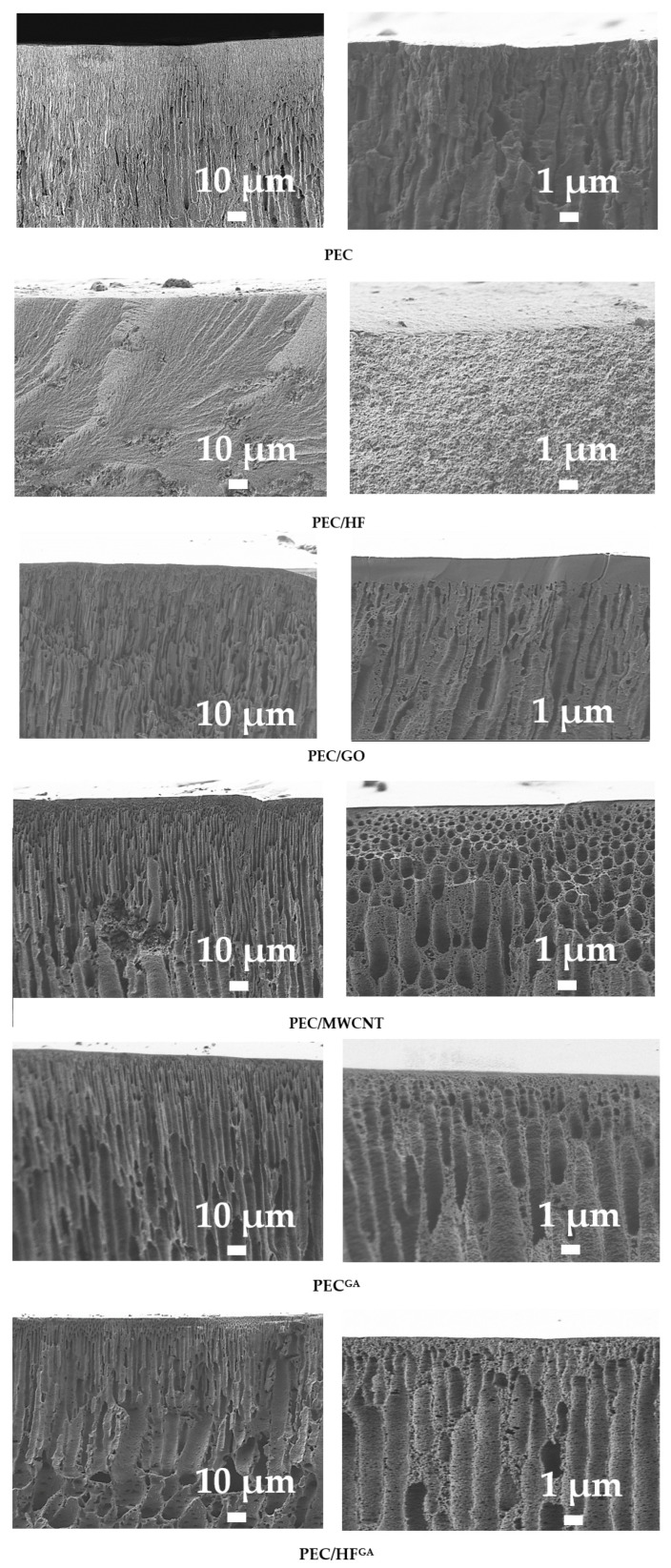
SEM micrographs of cross-sections for membranes based on PEC and PEC/CP composite at different magnifications.

The surface and inner morphology of the membranes were studied using microscopic methods (SEM and AFM). Cross-sectional SEM micrographs of the PEC-based membranes are presented in Figure 10.

**Figure 11:**
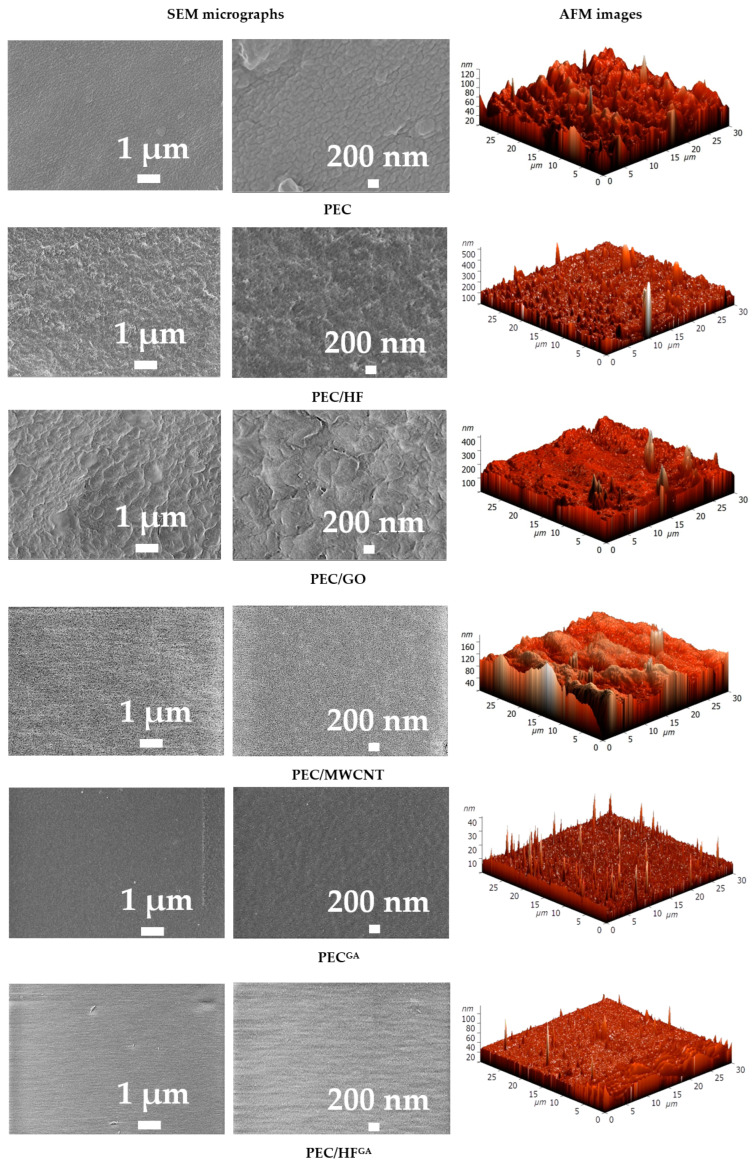
Surface SEM micrographs at different magnifications and AFM images of membranes based on PEC and PEC/CP composite.

All membranes showed an asymmetric structure (i.e., a structure consisting of an extremely thin top layer and a much thicker and highly porous layer) [60]. All membranes, except PEC/HF, have a dense upper layer with finger-like macrovoids. In most cases, the finger-like membrane morphology is formed by the process of instantaneous delamination [61]. The same effect has been observed for PEC (PSS/PEI)-based membranes in [16,17]. The introduction of CP such as GO and MWCNT into the PEC matrix leads to the formation of macrovoids with a larger and more “open structural” size [62], as well as to the compaction of the upper dense layer, especially for the PEC/GO membrane. This may be related to changes in the rate of formation of the PEC matrix due to the addition of modifiers with a large number of functional groups (namely, oxygen-containing groups) and their higher conductivity, as well as changes in both the pH and viscosity of the casting solutions during modification [63,64]. The PEC/HF membrane had a sponge-like structure with a large and complex pore tortuosity. The formation of a hydrogen bonding system between the HF and PEI (confirmed by FTIR data), due to the large number of functional groups (OH in HF and amine in PEI), can lead to delayed demixing [65] and eventually to the formation of a spongy internal structure of the PEC matrix [21]. The introduction of CP into the PEC matrix would allow the creation of nanoporous fragments in the membranes, increasing the surface area and providing additional channels for the transport of components (particularly water) and the retention of foulants [66]. Cross-linking the PEC membrane with GA (PEC^GA^ membrane) resulted in a more open porous structure with slight compaction of the top selective layer [16]. For the cross-linked PEC/HF^GA^ membrane, the most interesting effect was revealed when the cross-sectional structure was spongy-porous and consisted of finger-like macrovoids due to the combined effect of the modifier and cross-linking agent. Surface SEM micrographs and AFM images of the PEC-based membranes are presented in Figure 11.

The surface of the PEC membrane was free of defects and had a patterned structure due to the high degree of branching of the PEI used [17]. The introduction of CP into the PEC matrix resulted in the formation of a rougher surface of the modified membranes with different plastic deformations. GO results in a flaky surface structure, which may be due to the formation of a hydrogen bonding system between PEC and carboxyl groups of GO and its lamellar shape with a higher particle size range compared to other modifiers [67,68]. The PEC/MWCNT membrane is characterized by the presence of small, uniformly distributed pores on the surface. This can be explained by the high surface porosity structure of MWCNT [69], and the same effect was observed for the modified polyethersulfone membranes in [70]. A uniformly rough surface structure was observed for the PEC/HF membrane due to the smallest size of HF particles among all modifiers [21], which could also indicate a uniform distribution of particles in the PEC matrix. Cross-linking of PEC and PEC/HF membranes with GA resulted in a decrease in surface roughness, indicating the binding of polymer chains and functional groups of the modifier [42]. Based on AFM images, surface roughness was evaluated in terms of average (Ra) and root mean square parameters (Table 3). To evaluate the effect of the modifiers and cross-linking, the membrane surface was also evaluated by measuring the contact angles of water (Table 3).

The values of the surface parameters are in agreement with the SEM data. Modification of PEC with CP nanoparticles resulted in an increase in roughness in the following series of membranes: PEC/HF < PEC/MWCNT < PEC/GO. This dependence was also observed when modifying chitosan membranes in [21] due to their nature and particle size. Cross-linking of the membranes (PEC^GA^ and PEC/HF^GA^) resulted in lower surface roughness values [71]. It should also be noted that the introduction of CP into the PEC matrix resulted in surface hydrophilization (a reduction in the contact angle value). The lowest value for the modified membranes was observed for the PEC/HF membrane due to the largest number of polar hydroxyl groups of the modifier HF [72]. The contact angle value for the cross-linked PEC^GA^ membrane was close to that previously obtained for the PEC membrane prepared using PSS and PEI of a different molecular weight (25 kDa) [17].

**Figure 12:**
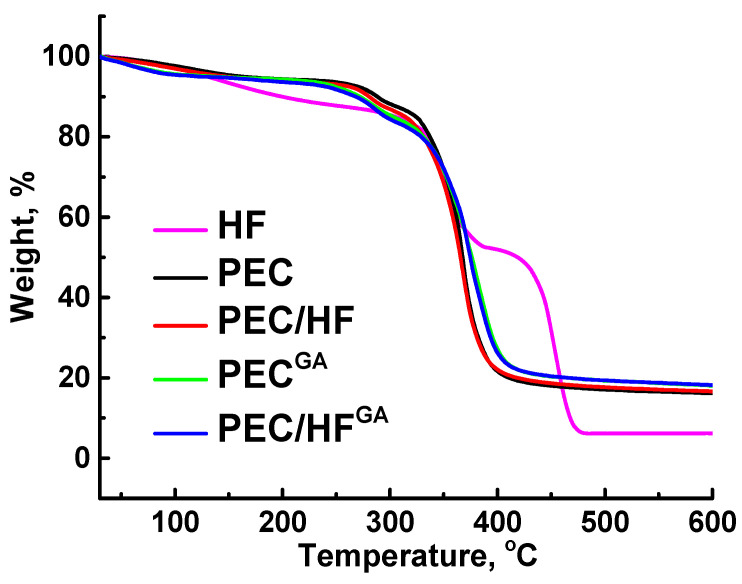
TGA curves of HF and developed untreated and cross-linked membranes based on PEC and PEC/HF(1%) composite.

The thermochemical properties of the developed membranes were investigated using the TGA method (Figure 12). This allows for the evaluation of the temperature range over which the membranes are expected to be used in industry. All PEC-based membranes showed high degradation onset temperatures (>350 °C) due to the ionic interaction of PSS and PEI [73], which stabilized the sulfonate and amine groups [73]. It is also worth noting that the degradation behavior of untreated and cross-linked membranes was different; cross-linked membranes had more residue upon degradation. Also, the introduction of even a small amount (1 wt.%) of HF resulted in greater stability of the modified composite membranes compared to the unmodified membranes due to the modifier’s high thermochemical stability up to 400 °C [74]. Thus, it has been shown that the developed membranes based on PEC (PSS/PEI) modified with HF are promising for industrial application even at elevated temperatures (up to 300 °C).

### 3.3. Theoretical Consideration

**Figure 13:**
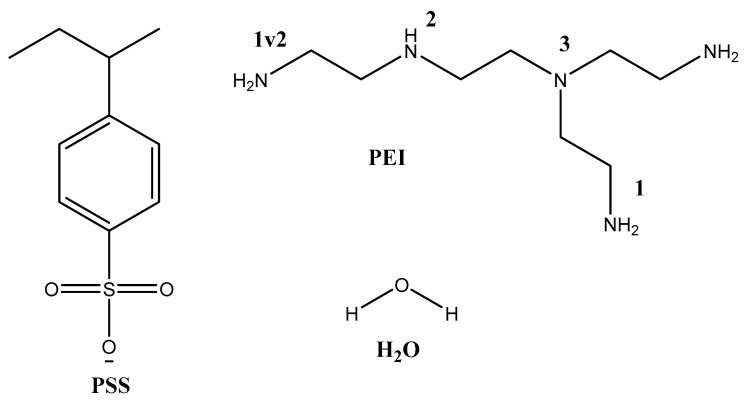
Model molecules and positions of amino groups.

The primary objective of the computational study was to investigate the interaction between PSS and PEI for the formation of PEC, as well as to assess the influence of PEI protonation on the properties of the developed membranes. To this end, the model molecules selected for examination included H_2_O, the PSS monomer, and branched PEI (composed of four monomeric fragments) featuring primary, secondary, and tertiary amine groups. Depending on the type of amino group being discussed, the corresponding interaction types were designated as PEI1, PEI1v2, PEI2, and PEI3 (Figure 13). Additionally, four forms of PEI were optimized during the course of the study: PEI1^+^, PEI1v2^+^, PEI2^+^, and PEI3^+^, each protonated at different positions. A hydrogen atom was used as the terminal group. The XYZ coordinates are specified in Table S1 of the Supplementary Materials.

Following the optimization of the geometries of the initial model molecules in order to investigate potential interactions, several hypothetical associates were generated. The alteration in Gibbs free energy, as presented in Table 4, was determined by calculating the difference between the energy of the associate and that of the initial molecules.

**Figure 14:**
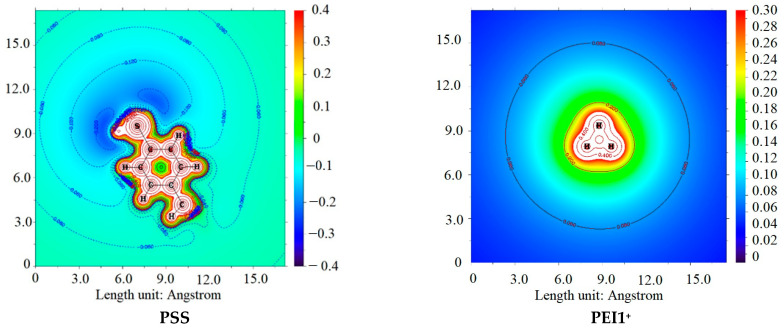

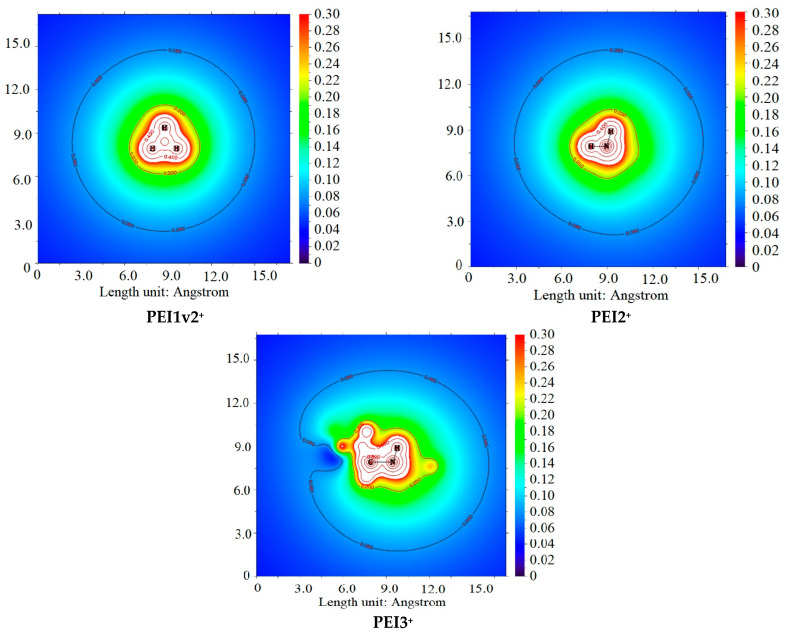
ESP plane plots of model PSS and PEI^+^ ions.

As demonstrated in Table 4, the classical effect of lower basicity of tertiary amines compared to secondary and primary amines, attributed to steric hindrance, was confirmed. Interestingly, despite the inductive effect of substituents in secondary amines, the interaction of primary amines appears to be similar to or even more energetically favorable, likely due to steric hindrance. This characteristic is even more pronounced when considering the monoprotonated form of PEI in interactions with H_2_O and PSS. This fact is consistent with the hard and soft acids and bases (HSAB) theory [75], where PSS and H_2_O can be regarded as hard bases, while PEI3^+^ acts as a soft acid. Conversely, primary and secondary amines serve as sources of molecular electrostatic potential (ESP) (Figure 14), a conclusion further supported by the calculated atomic charges presented in Table 5. Additionally, Table 5 includes the energies of the HOMO and LUMO orbitals.

The significant energetic gain observed in the interaction between PSS and PEI^+^ (Table 4) can be attributed to electrostatic interactions and electron density redistribution upon associate formation, involving HOMO(PSS)-LUMO(PEI^+^) orbital interactions. As observed in the series PEI1^+^, PEI1v2^+^, PEI2^+^, and PEI3^+^, the HOMO(PSS)-LUMO(PEI^+^) energy gaps are 3.00, 3.07, 2.30, and 1.55 eV, respectively. This observation may be indicative of molecular orbital interactions [78] and further confirms that, in the case of tertiary amines, the delocalization of electron density plays a more significant role compared to primary amines. Concurrently, the elevated charge values on the hydrogen atoms of primary amines substantiate the more pronounced electrostatic interactions observed in these systems.

**Figure 15:**
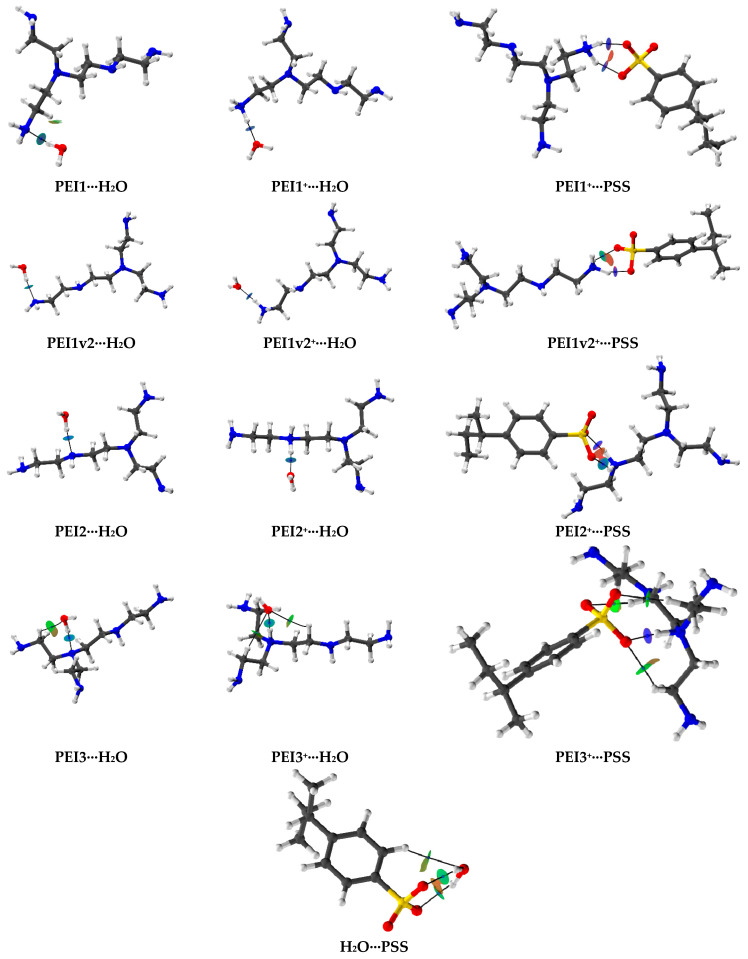
QTAIM distribution of BCPs and bond paths for the associates.

Additionally, during the study, the intermolecular interactions formed during the optimization of the associates were analyzed. A topological analysis (Bader quantum theory of atoms in molecules (QTAIM [79]) confirmed the non-covalent nature of these interactions. Figure 15 illustrates the bond critical points (blue spheres) and bond paths for the forming associates. To determine the type of these interactions, the non-covalent interaction plots (NCI plots [80]) were also presented in the images using an isovalue of 0.5 (e^1/3^ bohr)^−1^ and a color scale range of [−0.04, 0.02] e/bohr^3^. In these plots, the green color corresponds to van der Waals interactions, blue indicates hydrogen bonds, and red signifies repulsion.

Based on the obtained NCI plots, hydrogen bonds were identified as the predominant interaction type. To quantify the strength of such non-covalent interactions, bond orders were assessed. Table S2 in the Supplementary Materials presents the Wiberg bond indices (WBI) [81,82] and Fuzzy bond order (FBO [83]) values. The presented FBO values indicate that PSS exhibits a lower value (0.054) compared to PEI (0.107), suggesting the formation of stronger hydrogen bonds with water in the case of PEI. The interaction of PSS with PEI^+^ is characterized by high WBI values (>0.2), which may indicate a transfer of electron density and the formation of bonds with a borderline nature between non-covalent interactions and dative bonding [42]. This observation supports the possibility of PEC formation in this system.

### 3.4. Nanofiltration Performance Comparison

The nanofiltration performance of the developed PEC/HF^GA^ membrane was evaluated against PEC-based membranes reported in the literature, specifically for the filtration of anionic dye solutions under conditions close to those of this study (Table 6).

Differences in experimental conditions, permeability units, and membrane preparation methods make comparing PEC-based membranes quite challenging. However, the PEC/HF^GA^ membranes developed in this study exhibit high performance levels, such as improved permeability or rejection compared to others. Therefore, this developed membrane shows great promise for industrial applications in nanofiltration for water purification.

## 4. Conclusions

Nanofiltration membranes from PEC of PSS/PEI with improved performance were developed using a pH-induced APS method through modification with carbon nanoparticles such as GO, HF, and MWCNT for wastewater treatment of food anionic dyes and heavy metal ions.

The monomer ratio of PSS/PEI (1:1.5, 1:1.75, and 1:2) in the casting solution during membrane preparation was varied. An increase in the PEI content within the PEC matrix resulted in enhanced membrane permeability and a reduction in dye rejection coefficients due to the increase in the positive surface charge and the formation of a more open porous structure (confirmed by SEM). The PEC (PSS/PEI 1:1.75) membrane was chosen as optimal for further modification with CP (1 wt.% GO, HF, and MWCNT) because of its permeability and rejection ratios. This modification led to an increase in permeability and dye rejection coefficients, attributed to structural changes, surface hydrophilization, and the enhancement of the negative charge on the membrane surface due to the oxygen-containing functional groups of CP. The PEC/HF membrane exhibited the highest permeability among all membranes, attributed to its spongy-like inner structure (confirmed by SEM), which increased the effective contact area (confirmed by AFM), along with a highly hydrophilic surface (confirmed by contact angle measurements) due to a greater number of functional HF groups that facilitated water penetration. This PEC/HF (1 wt.%) membrane was further cross-linked with GA, which resulted in a more than 2-fold increase in permeability compared to the pristine PEC membrane and the highest dye retention (99.99 wt.%). This membrane was also tested in the nanofiltration of aqueous solutions of heavy metal ions (Cu^2+^, Cd^2+^, and Pb^2+^), where it demonstrated stable permeability (the membranes were not contaminated) and rejection coefficients exceeding 97%. Thus, it was demonstrated that the developed nanofiltration membrane is promising for industrial wastewater purification from dyes and heavy metal ions, even at elevated temperatures (confirmed by TGA).

## Figures and Tables

**Table 1:** Denotations of PEC-based membranes from PSS/PEI.

Membrane	PSS:PEI Ratio	CP Content, wt.%	Cross-Linking
PEC	1:1.75	-	-
PEC/HF		1	-
PEC/GO		1	-
PEC/MWCNT		1	-
PEC^GA^		-	0.01% GA
PEC/HF^GA^		1	0.01% GA

**Table 2:** Main characteristics of dyes.

	Structure	Molar Mass, g/mol	Wavelength Corresponding to Maximum Absorbance, nm
Sunset yellow (SY, E110)	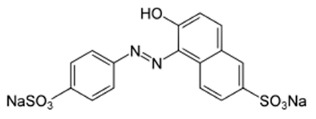	452.37	483
Congo red (CR, E129)	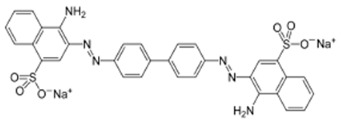	696.67	505
Alphazurine (AZ, E133)	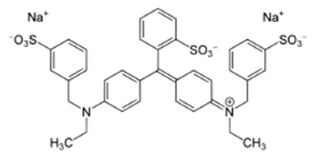	690.80	628

**Table 3:** Surface roughness and water contact angle of membranes.

Membranes	Surface Parameters		Water Contact Angle, °
	Ra, nm	Rq, nm	
PEC	8.5	11.0	70 ± 2
PEC/GO	16.3	24.9	64 ± 2
PEC/MWCNT	14.4	23.0	63 ± 2
PEC/HF	13.9	22.2	61 ± 2
PEC^GA^	4.3	13.8	42 ± 2
PEC/HF^GA^	2.22	4.1	39 ± 2

**Table 4:** The changes in the isothermal–isobaric potential during the association of components.

∆G (kJ/mol)									
B3LYP/6-311++G(d,p)	PSS	PEI1^+^	PEI1v2^+^	PEI2^+^	PEI3^+^	PEI1	PEI1v2	PEI2	PEI3
**PSS**	~	−410.3	−397.1	−383.6	−356.2				
**H_2_O**	−16.0	−35.8	−36.5	−20.3	−9.0	3.1	7.0	7.1	17.8

**Table 5:** Atomic dipole moment-corrected Hirshfeld (ADCH [76]) and restrained electrostatic potential (RESP [77]) atomic charges and energy of PSS HOMO and PEI^+^ LUMO.

B3LYP/6-311++G(d,p)	Atom	ADCH	RESP	E, eV
PSS	O^1^	−0.53	−0.71	−2.40
	O^2^	−0.53	−0.71	
	O^3^	−0.52	−0.71	
PEI1^+^	H^1^	0.30	0.38	−5.40
	H^2^	0.28	0.35	
	H^3^	0.28	0.36	
PEI1v2^+^	H^1^	0.31	0.38	−5.47
	H^2^	0.29	0.34	
	H^3^	0.28	0.34	
PEI2^+^	H^1^	0.26	0.24	−4.70
	H^2^	0.26	0.25	
PEI3^+^	H	0.24	0.22	−3.95

**Table 6:** Performance of PEC-based membranes in nanofiltration of anionic dye solutions.

PEC Membranes	Water Permeability/Permeance	Dye	Molar Mass, g/mol	Rejection Coefficient, %	Ref.
PSS/PEI/HF^GA^	9.90 kg/(m^2^ h atm)	SY	452	99.99	This study
		CR	697	99.99	
		AZ	691	99.99	
PEI-modified GO/polyacrylic acid	0.84 kg/(m^2^ h bar)	CR	697	99.5	[9]
quaternary ammonium cellulose ether/sodium carboxymethyl cellulose)	1.9 L/(m^2^ h bar)	XO *	673	99.3	[84]
PSS/PDADMAC	6.2 L/(m^2^ h bar)	MO *	327	91.7	[85]
chitosan/dextran sulfate sodium	6.7 L/(m^2^ h bar)	MB *	800	99.5	[86]
polyether ether ketone (SPEEK)/PEI	20 L/(m^2^ h bar)	CR	697	99	[87]
		MO *	327	99	
SPEEK/PDADMAC	~27 L/(m^2^ h bar)	MO *	327	~93	[88]
PEI/sodium alginate	57.4 L/(m^2^ h bar)	CR	697	99.4	[89]

* XO—xylenol orange; MO—methyl orange; MB—methyl blue.

## Data Availability

Data are contained within the article and Supplementary Materials.

## Supplementary Materials

The following supporting information can be downloaded at: https://www.mdpi.com/article/10.3390/polym17101306/s1, Figure S1: (**a**) Permeability and (**b**) rejection coefficient of dyes for PEC/HF (0–5 wt.%) membranes; Figure S2: ^13^C NMR spectra of (**a**) PSS, (**b**) PEI, and (**c**) HF; Table S1: The Cartesian coordinates of molecules and their associates; Table S2: WBI and FBO values, and the interaction lengths (d).

## References

[1] Magni M., Jones E.R., Bierkens M.F.P., van Vliet M.T.H. (2025). Global energy consumption of water treatment technologies. Water Res..

[2] Zhang B., Kong L., Yan X., Zhang H., Wang Z., Xia S., Han Z., Xin Y., Ding A., Ma J. (2025). Recent progress in graphitic carbon nitride-based catalysts for water treatment: Contaminant elimination, disinfection and membrane applications. Sep. Purif. Technol..

[3] Dmitrenko M., Kuzminova A., Zolotarev A., Selyutin A., Ermakov S., Penkova A. (2023). Nanofiltration Mixed Matrix Membranes from Cellulose Modified with Zn-Based Metal—Organic Frameworks for the Enhanced Water Treatment from Heavy Metal Ions. Polymers.

[4] Alhussaini M.A., Souza-Chaves B.M., Felix V., Achilli A. (2024). Comparative analysis of reverse osmosis and nanofiltration for the removal of dissolved contaminants in water reuse applications. Desalination.

[5] Zheng J., Li Y., Xu D., Zhao R., Liu Y., Li G., Gao Q., Zhang X., Volodine A., Van der Bruggen B. (2022). Facile fabrication of a positively charged nanofiltration membrane for heavy metal and dye removal. Sep. Purif. Technol..

[6] Mikulášek P., Cuhorka J. (2010). Nanofiltration in the manufacture of liquid dyes production. Water Sci. Technol..

[7] Durmaz E.N., Willott J.D., Mizan M.M.H., de Vos W.M. (2021). Tuning the charge of polyelectrolyte complex membranes prepared via aqueous phase separation. Soft Matter.

[8] Wang Y.-C., Kumar S.R., Shih C.-M., Hung W.-S., An Q.-F., Hsu H.-C., Huang S.-H., Lue S.J. (2017). High permeance nanofiltration thin film composites with a polyelectrolyte complex top layer containing graphene oxide nanosheets. J. Membr. Sci..

[9] Wang N., Ji S., Zhang G., Li J., Wang L. (2012). Self-assembly of graphene oxide and polyelectrolyte complex nanohybrid membranes for nanofiltration and pervaporation. Chem. Eng. J..

[10] Dmitrenko M.E., Kuzminova A.I., Zolotarev A.A., Korniak A.S., Ermakov S.S., Su R., Penkova A.V. (2022). Novel mixed matrix membranes based on polyelectrolyte complex modified with fullerene derivatives for enhanced pervaporation and nanofiltration. Sep. Purif. Technol..

[11] Zhao F.Y., An Q.F., Ji Y.L., Gao C.J. (2015). A novel type of polyelectrolyte complex/MWCNT hybrid nanofiltration membranes for water softening. J. Membr. Sci..

[12] Gan L., Zhang J., Wu Y., Chen Z., Zhao Z., Lin S., Jiang Y. (2025). Tailoring Polyelectrolyte Multilayer Nanofiltration Membranes by Aerosol-Assisted Printing: Insights into Membrane Formation Mechanisms. Environ. Sci. Technol..

[13] Korzhova E., Déon S., Koubaa Z., Fievet P., Lopatin D., Baranov O. (2020). Modification of commercial UF membranes by electrospray deposition of polymers for tailoring physicochemical properties and enhancing filtration performances. J. Membr. Sci..

[14] Liang Y., Lin S. (2020). Intercalation of zwitterionic surfactants dramatically enhances the performance of low-pressure nanofiltration membrane. J. Membr. Sci..

[15] Alghamdi A.M. (2021). Fast and Versatile Pathway in Fabrication of Polyelectrolyte Multilayer Nanofiltration Membrane with Tunable Properties. J. Chem..

[16] Baig M.I., Sari P.P.I., Li J., Willott J.D., de Vos W.M. (2021). Sustainable Aqueous Phase Separation membranes prepared through mild pH shift induced polyelectrolyte complexation of PSS and PEI. J. Membr. Sci..

[17] Haque Mizan M.M., Rastgar M., Aktij S.A., Asad A., Karami P., Rahimpour A., Sadrzadeh M. (2023). Organic solvent-free polyelectrolyte complex membrane preparation: Effect of monomer mixing ratio and casting solution temperature. J. Membr. Sci..

[18] Abdelhalim A.O.E., Sharoyko V.V., Meshcheriakov A.A., Luttsev M.D., Potanin A.A., Iamalova N.R., Zakharov E.E., Ageev S.V., Petrov A.V., Vasina L.V. (2020). Synthesis, characterisation and biocompatibility of graphene—L-methionine nanomaterial. J. Mol. Liq..

[19] Semenov K.N., Charykov N.A., Keskinov V.N. (2011). Fullerenol Synthesis and Identification. Properties of the Fullerenol Water Solutions. J. Chem. Eng. Data.

[20] Marcos M.A., Podolsky N.E., Cabaleiro D., Lugo L., Zakharov A.O., Postnov V.N., Charykov N.A., Ageev S.V., Semenov K.N. (2019). MWCNT in PEG-400 nanofluids for thermal applications: A chemical, physical and thermal approach. J. Mol. Liq..

[21] Dmitrenko M., Mikhailovskaya O., Kuzminova A., Mazur A., Su R., Penkova A. (2024). Pervaporation chitosan membranes modified with carbon nanoparticles for enhanced isopropanol dehydration. J. Mater. Sci..

[22] Zeng X., Zhou G., Xu Q., Xiong Y., Luo C., Wu J. (2010). A new technique for dispersion of carbon nanotube in a metal melt. Mater. Sci. Eng. A.

[23] Munkhbayar B., Nine M.J., Hwang S., Kim J., Bae K., Chung H., Jeong H. (2012). Effect of grinding speed changes on dispersibility of the treated multi-walled carbon nanotubes in aqueous solution and its thermal characteristics. Chem. Eng. Process. Process Intensif..

[24] Kukovecz Á., Kanyó T., Kónya Z., Kiricsi I. (2005). Long-time low-impact ball milling of multi-wall carbon nanotubes. Carbon.

[25] Lindén J.B., Larsson M., Kaur S., Skinner W.M., Miklavcic S.J., Nann T., Kempson I.M., Nydén M. (2015). Polyethyleneimine for copper absorption II: Kinetics, selectivity and efficiency from seawater. RSC Adv..

[26] Baker R.W. (2000). Membrane Technology and Applications.

[27] Dmitrenko M., Sushkova X., Chepeleva A., Liamin V., Mikhailovskaya O., Kuzminova A., Semenov K., Ermakov S., Penkova A. (2023). Modification Approaches of Polyphenylene Oxide Membranes to Enhance Nanofiltration Performance. Membranes.

[28] Frisch M.J. (2016). Gaussian 16.

[29] Becke A.D. (1992). Density-functional thermochemistry. I. The effect of the exchange-only gradient correction. J. Chem. Phys..

[30] Becke A.D. (1988). Density-functional exchange-energy approximation with correct asymptotic behavior. Phys. Rev. A.

[31] Lee C., Yang W., Parr R.G. (1988). Development of the Colle-Salvetti correlation-energy formula into a functional of the electron density. Phys. Rev. B.

[32] Lu T., Chen F. (2012). Multiwfn: A multifunctional wavefunction analyzer. J. Comput. Chem..

[33] Humphrey W., Dalke A., Schulten K. (1996). VMD: Visual molecular dynamics. J. Mol. Graph..

[34] Vaysizadeh A., Zinatizadeh A.A., Zinadini S. (2021). Fouling mitigation and enhanced dye rejection in UF and NF membranes via layer-by-layer (LBL) assembly and altering PVP percentage as pore former. Environ. Technol. Innov..

[35] Gholami N., Mahdavi H. (2018). Nanofiltration composite membranes of polyethersulfone and graphene oxide and sulfonated graphene oxide. Adv. Polym. Technol..

[36] Lee A., Beak S., Lee S., Kim G., Lee D., Kim S., Sung Y., Jeong H. (2020). Hydrophilic/Hydrophobic characteristics on the carbon nanotube buckypapers with various mechanical and chemical manufacture process. Diam. Relat. Mater..

[37] Wang F., Tarabara V.V. (2008). Pore blocking mechanisms during early stages of membrane fouling by colloids. J. Colloid Interface Sci..

[38] Dmitrenko M., Liamin V., Kuzminova A., Mazur A., Lahderanta E., Ermakov S., Penkova A. (2020). Novel Mixed Matrix Sodium Alginate–Fullerenol Membranes: Development, Characterization, and Study in Pervaporation Dehydration of Isopropanol. Polymers.

[39] Wanjiya M., Zhang J.-C., Wu B., Yin M.-J., An Q.-F. (2024). Nanofiltration membranes for sustainable removal of heavy metal ions from polluted water: A review and future perspective. Desalination.

[40] Grenda K., Idström A., Evenäs L., Persson M., Holmberg K., Bordes R. (2022). An analytical approach to elucidate the architecture of polyethyleneimines. J. Appl. Polym. Sci..

[41] Devi D.A., Smitha B., Sridhar S., Jawalkar S.S., Aminabhavi T.M. (2007). Novel sodium alginate/polyethyleneimine polyion complex membranes for pervaporation dehydration at the azeotropic composition of various alcohols. J. Chem. Technol. Biotechnol..

[42] Dmitrenko M., Mikhailovskaya O., Dubovenko R., Kuzminova A., Myznikov D., Mazur A., Semenov K., Rusalev Y., Soldatov A., Ermakov S. (2024). Pervaporation Membranes Based on Polyelectrolyte Complex of Sodium Alginate/Polyethyleneimine Modified with Graphene Oxide for Ethanol Dehydration. Polymers.

[43] Dai F., Zhuang Q., Huang G., Deng H., Zhang X. (2023). Infrared Spectrum Characteristics and Quantification of OH Groups in Coal. ACS Omega.

[44] Xu J., Cui X., Wang H., Li J., Dong S. (2011). Preparation of ultrafine poly(sodium 4-styrenesulfonate) fibres via electrospinning. Bull. Mater. Sci..

[45] Ravelo-Nieto E., Duarte-Ruiz A., Reyes L.H., Cruz J.C. Synthesis and Characterization of a Fullerenol Derivative for Potential Biological Applications. Proceedings of the 2nd International Online-Conference on Nanomaterials.

[46] Yang X., Zhen M., Li G., Liu X., Wang X., Shu C., Jiang L., Wang C. (2013). Preparation of Pd-decorated fullerenols on carbon nanotubes with excellent electrocatalytic properties in alkaline media. J. Mater. Chem. A.

[47] Abdelhalim A.O.E., Sharoyko V.V., Meshcheriakov A.A., Martynova S.D., Ageev S.V., Iurev G.O., Al Mulla H., Petrov A.V., Solovtsova I.L., Vasina L.V. (2020). Reduction and functionalization of graphene oxide with L-cysteine: Synthesis, characterization and biocompatibility. Nanomed. Nanotechnol. Biol. Med..

[48] Marcano D.C., Kosynkin D.V., Berlin J.M., Sinitskii A., Sun Z., Slesarev A., Alemany L.B., Lu W., Tour J.M. (2010). Improved Synthesis of Graphene Oxide. ACS Nano.

[49] Sarode V.B., Patil R.D., Chaudhari G.E. Characterization of functionalized multi-walled carbon nanotubes. Mater. Today Proc..

[50] Sbai K., Rahmani A., Chadli H., Bantignies J.-L., Hermet P., Sauvajol J.-L. (2006). Infrared Spectroscopy of Single-Walled Carbon Nanotubes. J. Phys. Chem. B.

[51] Shen J.-N., Chu Y.-X., Ruan H.-M., Wu L.-G., Gao C.-J., Van der Bruggen B. (2014). Pervaporation of benzene/cyclohexane mixtures through mixed matrix membranes of chitosan and Ag+/carbon nanotubes. J. Membr. Sci..

[52] Ong Y.T., Ahmad A.L., Zein S.H.S., Sudesh K., Tan S.H. (2011). Poly(3-hydroxybutyrate)-functionalised multi-walled carbon nanotubes/chitosan green nanocomposite membranes and their application in pervaporation. Sep. Purif. Technol..

[53] Qiu S., Wu L., Shi G., Zhang L., Chen H., Gao C. (2010). Preparation and pervaporation property of chitosan membrane with functionalized multiwalled carbon nanotubes. Ind. Eng. Chem. Res..

[54] Yeang Q.W., Zein S.H.S., Sulong A.B., Tan S.H. (2013). Comparison of the pervaporation performance of various types of carbon nanotube-based nanocomposites in the dehydration of acetone. Sep. Purif. Technol..

[55] Sivashankari P.R., Krishna Kumar K., Devendiran M., Prabaharan M. (2021). Graphene oxide-reinforced pectin/chitosan polyelectrolyte complex scaffolds. J. Biomater. Sci. Polym. Ed..

[56] Lefay C., Guillaneuf Y., Moreira G., Thevarajah J.J., Castignolles P., Ziarelli F., Bloch E., Major M., Charles L., Gaborieau M. (2013). Heterogeneous modification of chitosan via nitroxide-mediated polymerization. Polym. Chem..

[57] Chowdhury S.I., Tanaka R., Nakayama Y., Shiono T. (2019). Copolymerization of Norbornene and Styrene with Anilinonaphthoquinone-Ligated Nickel Complexes. Polymers.

[58] Wei Y., Yu X., Zhang L., Liu W., Yong C., Yun T. (2024). Synthesis and function studies on formaldehyde adsorption of PEI-PU copolymers. J. Phys. Conf. Ser..

[59] Gutowski W.S., Bilyk A., Li S., Espiritu M., Burgar I. (2005). The influence of structure of the interface and interphase on paint adhesion. Compos. Interfaces.

[60] Stewart M.I. (2014). Gas Sweetening. Surface Production Operations.

[61] Koenhen D.M., Mulder M.H.V., Smolders C.A. (1977). Phase separation phenomena during the formation of asymmetric membranes. J. Appl. Polym. Sci..

[62] Yu J., Boudjelida S., Galiano F., Figoli A., Bonchio M., Carraro M. (2022). Porous Polymeric Membranes Doped with Halloysite Nanotubes and Oxygenic Polyoxometalates. Adv. Mater. Interfaces.

[63] Lin C.-H., Chien M.-Y., Chuang Y.-C., Lai C.-C., Sun Y.-M., Liu T.-Y. (2022). Porous Membranes of Polysulfone and Graphene Oxide Nanohybrids for Vanadium Redox Flow Battery. Polymers.

[64] Kuzminova A., Dmitrenko M., Dubovenko R., Puzikova M., Mikulan A., Korovina A., Koroleva A., Selyutin A., Semenov K., Su R. (2024). Development and Study of Novel Ultrafiltration Membranes Based on Cellulose Acetate. Polymers.

[65] Kamp J., Emonds S., Borowec J., Restrepo Toro M.A., Wessling M. (2021). On the organic solvent free preparation of ultrafiltration and nanofiltration membranes using polyelectrolyte complexation in an all aqueous phase inversion process. J. Membr. Sci..

[66] Regmi C., Kshetri Y.K., Wickramasinghe S.R. (2024). Carbon-Based Nanocomposite Membranes for Membrane Distillation: Progress, Problems and Future Prospects. Membranes.

[67] Qian X., Li N., Wang Q., Ji S. (2018). Chitosan/graphene oxide mixed matrix membrane with enhanced water permeability for high-salinity water desalination by pervaporation. Desalination.

[68] Kim J., Park S., Choi M., Kim S., Heo J., Yeom E., Kim S., Lee H., Kim S. (2023). Simply controlling the surface structure of graphene oxide films using multiple drop-casting. Diam. Relat. Mater..

[69] Li J., Wang Q., Deng L., Kou X., Tang Q., Hu Y. (2020). Fabrication and characterization of carbon nanotubes-based porous composite forward osmosis membrane: Flux performance, separation mechanism, and potential application. J. Membr. Sci..

[70] Ngoma M.M., Mathaba M., Moothi K. (2021). Effect of carbon nanotubes loading and pressure on the performance of a polyethersulfone (PES)/carbon nanotubes (CNT) membrane. Sci. Rep..

[71] Grover C.N., Gwynne J.H., Pugh N., Hamaia S., Farndale R.W., Best S.M., Cameron R.E. (2012). Crosslinking and composition influence the surface properties, mechanical stiffness and cell reactivity of collagen-based films. Acta Biomater..

[72] Dmitrenko M.E., Penkova A.V., Kuzminova A.I., Atta R.R., Zolotarev A.A., Mazur A.S., Vezo O.S., Lahderanta E., Markelov D.A., Ermakov S.S. (2019). Development and investigation of novel polyphenylene isophthalamide pervaporation membranes modified with various fullerene derivatives. Sep. Purif. Technol..

[73] Li J., Krishna B.A., van Ewijk G., van Dijken D.J., de Vos W.M., van der Gucht J. (2022). A comparison of complexation induced brittleness in PEI/PSS and PEI/NaPSS single-step coatings. Colloids Surf. A Physicochem. Eng. Asp..

[74] Koruga D., Stanković I., Matija L., Kuhn D., Christ B., Dembski S., Jevtić N., Janać J., Pavlović V., De Wever B. (2024). Comparative Studies of the Structural and Physicochemical Properties of the First Fullerene Derivative FD-C60 (Fullerenol) and Second Fullerene Derivate SD-C60 (3HFWC). Nanomaterials.

[75] Pearson R.G. (1963). Hard and Soft Acids and Bases. J. Am. Chem. Soc..

[76] Lu T., Chen F. (2012). Atomic Dipole Moment Corrected Hirshfeld Population Method. J. Theor. Comput. Chem..

[77] Bayly C.I., Cieplak P., Cornell W., Kollman P.A. (1993). A well-behaved electrostatic potential based method using charge restraints for deriving atomic charges: The RESP model. J. Phys. Chem..

[78] Cheranyova A.M., Zelenkov L.E., Baykov S.V., Izotova Y.A., Ivanov D.M., Bokach N.A., Kukushkin V.Y. (2024). Intermolecular Metal-Involving Pnictogen Bonding: The Case of σ-(SbIII)-Hole···dz2 [PtII] Interaction. Inorg. Chem..

[79] Bader R.F.W. (1990). Atoms in Molecules.

[80] Johnson E.R., Keinan S., Mori-Sánchez P., Contreras-García J., Cohen A.J., Yang W. (2010). Revealing Noncovalent Interactions. J. Am. Chem. Soc..

[81] Wiberg K.B. (1968). Application of the pople-santry-segal CNDO method to the cyclopropylcarbinyl and cyclobutyl cation and to bicyclobutane. Tetrahedron.

[82] Trindle C. (1969). Bond index description of delocalization. J. Am. Chem. Soc..

[83] Mayer I., Salvador P. (2004). Overlap populations, bond orders and valences for ‘fuzzy’ atoms. Chem. Phys. Lett..

[84] Ji Y., An Q., Zhao Q., Chen H., Qian J., Gao C. (2010). Fabrication and performance of a new type of charged nanofiltration membrane based on polyelectrolyte complex. J. Membr. Sci..

[85] Hung S.-H.J., Chiang M.-C., Schiffman J.D. (2025). Optimization of Polyelectrolyte Coacervate Membranes via Aqueous Phase Separation. ACS Appl. Mater. Interfaces.

[86] Ye C.-C., Zhao F.-Y., Wu J.-K., Weng X.-D., Zheng P.-Y., Mi Y.-F., An Q.-F., Gao C.-J. (2017). Sulfated polyelectrolyte complex nanoparticles structured nanoflitration membrane for dye desalination. Chem. Eng. J..

[87] Li C., Hu D., Liu L., Zhu L., Xu M., Wang C., Li Y. (2022). Positively charged loose nanofiltration membranes prepared by a green ionic cross-link method. J. Mater. Sci..

[88] Li X., Goyens W., Ahmadiannamini P., Vanderlinden W., De Feyter S., Vankelecom I. (2010). Morphology and performance of solvent-resistant nanofiltration membranes based on multilayered polyelectrolytes: Study of preparation conditions. J. Membr. Sci..

[89] Zhang W.-H., Liu Z.-J., Yin M.-J., Ren Y.-H., Jin C.-G., Wang N., An Q.-F. (2022). Fabrication of stable polyelectrolyte complexed membrane for dye/salt separation via dynamic self-assembly coupled ice-templating technique. Desalination.

