# Supplementary material for: Nanofiltration Membranes from Poly(sodium-p-styrenesulfonate)/Polyethylenimine Polyelectrolyte Complex Modified with Carbon Nanoparticles for Enhanced Water Treatment
Source: Polymers (Basel). 2025 May 10;17(10):1306. doi: 10.3390/polym17101306 (PMC12114722; doi:10.3390/polym17101306)
Supplement: Supplementary file 1 [file polymers-17-01306-s001.zip › polymers-3612249-supplementary.pdf]

Nanofiltration membranes from PSS/PEI polyelectrolyte complex modified with carbon nanoparticles for enhanced water treatment

Mariia Dmitrenko [1,*], Olga Mikhailovskaya [1], Roman Dubovenko [1], Anton Mazur [1], Anna Kuzminova [1], Igor Prikhodko [1], Konstantin Semenov [2], Rongxin Su [3] and Anastasia Penkova [1,*]

[1]St. Petersburg State University, 7/9 Universitetskaya nab., St. Petersburg 199034, Russia;
2Pavlov First Saint Petersburg State Medical University, L'va Tolstogo ulitsa 6–8, St. Petersburg 197022, Russia;
3State Key Laboratory of Chemical Engineering, School of Chemical Engineering and Technology, Tianjin University, Tianjin 300072, China
* Correspondence: Correspondence: m.dmitrienko@spbu.ru (M.D.), a.penkova@spbu.ru (A.P.), Tel.: +7(812)363-60-00 (ext. 3367)

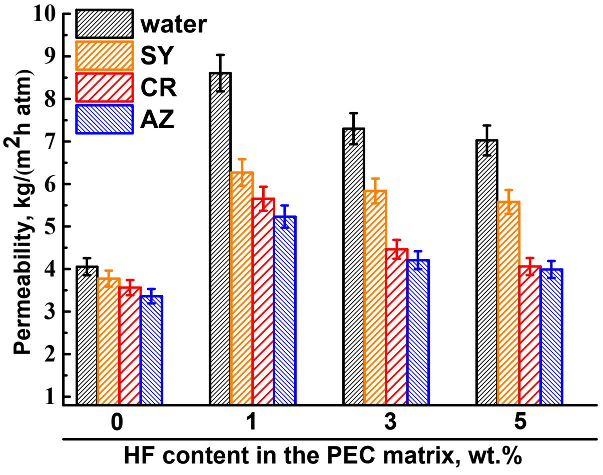

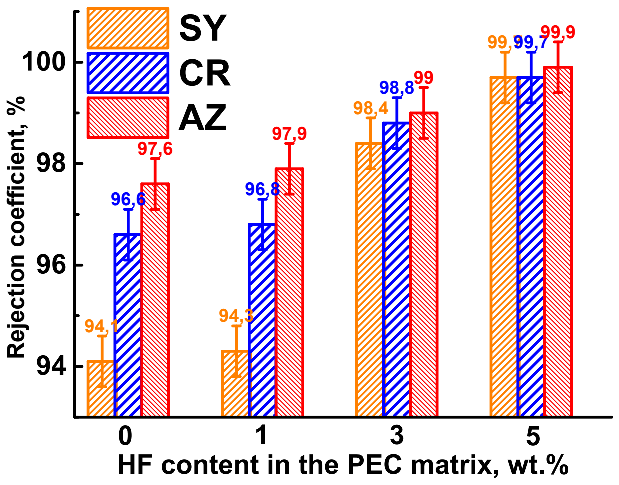

(a) (b)

Figure S1. (a) Permeability and **(b)** rejection coefficient of dyes for PEC/HF (0-5 wt.%) membranes.

(a)

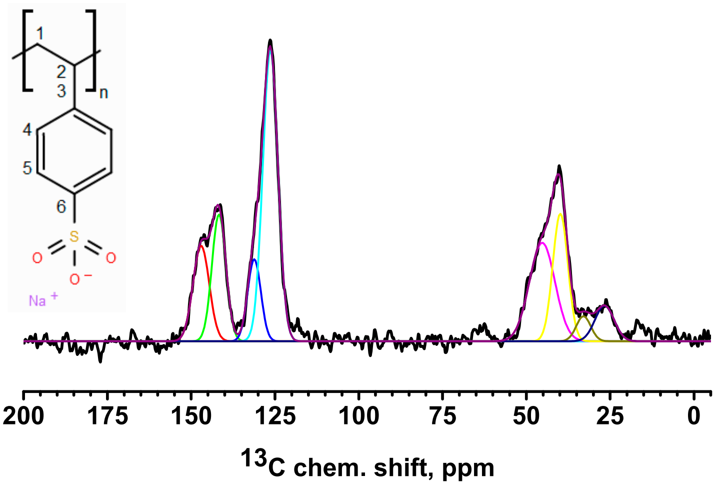

(b)

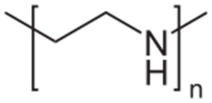

(c)

Figure S2. ^{13}C NMR spectra of **(a)** PSS, **(b)** PEI and **(c)** HF.

Table S1. The Cartesian coordinates of molecules and their associates.

Atom	X	Y	Z	Atom	X	Y	Z
	PSS				*PSS···H₂O*		
**C**	3.63858	2.02776	-0.34204	**O**	-3.59671	2.73448	0.23762
**C**	3.91066	0.53757	-0.56390	**H**	-3.59759	2.13346	-0.52587
**C**	3.14191	-0.39451	0.39898	**H**	-3.46817	2.08206	0.94610
**C**	3.60488	-1.85344	0.22727	**C**	3.92572	-0.13701	2.05368
**C**	1.63007	-0.26498	0.26959	**C**	4.12980	0.61164	0.73393
**C**	0.96950	-0.56388	-0.93059	**C**	3.47014	-0.06823	-0.48646
**C**	-0.41345	-0.44729	-1.04033	**C**	3.86921	0.64849	-1.79000
**C**	-1.17272	-0.03027	0.05314	**C**	1.95817	-0.18829	-0.35163
**C**	-0.53386	0.27261	1.25133	**C**	1.14205	0.94733	-0.23771
**C**	0.85331	0.15521	1.35335	**C**	-0.23931	0.83474	-0.12092
**S**	-2.99626	0.06220	-0.07769	**C**	-0.83697	-0.42708	-0.11713
**O**	-3.23583	0.56524	-1.45480	**C**	-0.04757	-1.56477	-0.23103
**O**	-3.44469	-1.33801	0.13931	**C**	1.33893	-1.44026	-0.34653
**O**	-3.39266	1.00106	1.00316	**S**	-2.64863	-0.56781	0.03860

**H**	4.21347	2.64212	-1.04211	**O**	-2.96116	0.10191	1.33518
**H**	2.57997	2.26043	-0.47818	**O**	-3.18127	0.18786	-1.13289
**H**	3.91598	2.33162	0.67317	**O**	-2.92503	-2.02087	0.01250
**H**	4.98504	0.34202	-0.45041	**H**	4.42304	0.37964	2.88039
**H**	3.65960	0.26984	-1.59713	**H**	2.86485	-0.22384	2.29945
**H**	3.40179	-0.08663	1.42048	**H**	4.33695	-1.15081	1.99895
**H**	3.09044	-2.51137	0.93231	**H**	5.20444	0.71160	0.53360
**H**	4.68402	-1.94503	0.39470	**H**	3.74137	1.63251	0.82850
**H**	3.38978	-2.21914	-0.78136	**H**	3.86748	-1.09042	-0.53950
**H**	1.53980	-0.88799	-1.79683	**H**	3.43407	0.14903	-2.65939
**H**	-0.91717	-0.66338	-1.97532	**H**	4.95810	0.66341	-1.91070
**H**	-1.13227	0.61292	2.08826	**H**	3.51725	1.68445	-1.79463
**H**	1.34176	0.40029	2.29328	**H**	1.58785	1.93771	-0.23930
PEI				**H**	-0.85852	1.72045	-0.03255
**N**	-3.51979	-3.13055	-0.18125	**H**	-0.52965	-2.53498	-0.22603
**C**	-2.47604	-2.17008	-0.56161	**H**	1.94891	-2.33574	-0.43311
**C**	-2.48065	-0.98746	0.40936	***H_2O···PEI1***			
**N**	-1.43451	-0.00498	0.11363	**N**	3.60696	-2.07906	1.13109
**C**	-1.81876	1.35816	0.49046	**C**	2.39857	-1.23386	1.22808
**C**	-0.12880	-0.39267	0.65379	**C**	2.29849	-0.30606	0.01481
**C**	-2.70026	2.03764	-0.55970	**N**	1.09387	0.52948	0.05971
**N**	-3.08682	3.37566	-0.09431	**C**	1.28523	1.82991	-0.58901
**C**	1.03789	0.13107	-0.18578	**C**	-0.09354	-0.17760	-0.43365
**N**	2.31381	-0.23331	0.42749	**C**	1.96590	2.85379	0.32214
**C**	3.48202	0.18726	-0.34322	**N**	2.17041	4.11060	-0.40769
**C**	4.77702	-0.26499	0.32957	**C**	-1.38302	0.30583	0.23262
**N**	5.93820	0.16608	-0.45832	**N**	-2.54008	-0.38811	-0.32811
**H**	-3.63143	-3.84680	-0.89025	**C**	-3.81171	-0.00911	0.28319
**H**	-3.28175	-3.60304	0.68538	**C**	-4.97022	-0.80040	-0.32191
**H**	-2.70277	-1.79412	-1.56279	**N**	-6.23720	-0.41404	0.31188
**H**	-1.46943	-2.61145	-0.60850	**H**	3.64567	-2.72901	1.91026
**H**	-2.39684	-1.36018	1.44806	**H**	2.37171	-0.62428	2.14017
**H**	-3.45706	-0.50479	0.33751	**H**	1.53461	-1.90270	1.25863
**H**	-0.91933	1.96494	0.60992	**H**	2.35445	-0.90072	-0.91041
**H**	-2.33267	1.37153	1.47041	**H**	3.17325	0.35225	0.01655
**H**	-0.06066	-1.48194	0.67542	**H**	0.31241	2.23555	-0.87224
**H**	-0.01579	-0.05515	1.70188	**H**	1.86176	1.72750	-1.52760
**H**	-3.55768	1.39238	-0.80195	**H**	0.01294	-1.24418	-0.22756
**H**	-2.11826	2.14782	-1.47852	**H**	-0.18201	-0.08662	-1.53204
**H**	-3.54361	3.90006	-0.83219	**H**	2.89426	2.42892	0.73389
**H**	-3.74151	3.31716	0.67958	**H**	1.30646	3.05198	1.17135
**H**	0.93816	1.21911	-0.33978	**H**	2.49898	4.84404	0.21027
**H**	0.98608	-0.32914	-1.17740	**H**	2.86580	3.99981	-1.13921
**H**	2.36498	0.16891	1.35979	**H**	-1.46674	1.40330	0.14882
**H**	3.41239	-0.27581	-1.33539	**H**	-1.32863	0.07999	1.30225
**H**	3.52749	1.27675	-0.50593	**H**	-2.58307	-0.21710	-1.32935
**H**	4.85263	0.20748	1.31558	**H**	-3.74679	-0.23213	1.35560
**H**	4.72463	-1.35042	0.49655	**H**	-4.03314	1.06762	0.19776
**H**	6.80301	-0.01329	0.03976	**H**	-5.04820	-0.56239	-1.38891
**H**	5.98871	-0.34374	-1.33498	**H**	-4.73895	-1.87238	-0.24781
$PEI1^+$				**H**	-7.02596	-0.83410	-0.16696

**N**	-3.26885	-3.22522	-0.24546	**H**	-6.26992	-0.73340	1.27507
**C**	-2.34859	-2.04961	-0.59401	**H**	4.44522	-1.50657	1.19079
**C**	-2.41502	-0.96880	0.48800	**O**	3.35638	-3.44998	-1.41591
**N**	-1.41604	0.03819	0.19300	**H**	3.97102	-4.18489	-1.48928
**C**	-1.85301	1.41062	0.50853	**H**	3.52785	-3.04770	-0.53850
**C**	-0.08814	-0.29829	0.73705			***H_2O···PEI1v2***	
**C**	-2.74976	2.01839	-0.57393	**N**	-4.45721	-2.76349	-0.22283
**N**	-3.12446	3.37451	-0.18829	**C**	-3.26722	-1.97208	-0.56023
**C**	1.06272	0.24067	-0.11473	**C**	-3.13054	-0.79319	0.40711
**N**	2.33745	-0.16314	0.46527	**N**	-1.95228	0.03458	0.13154
**C**	3.49847	0.18764	-0.36038	**C**	-2.14315	1.43632	0.51416
**C**	4.80263	-0.24303	0.31047	**C**	-0.72288	-0.54012	0.68547
**N**	5.94226	0.12390	-0.53225	**C**	-2.89236	2.24493	-0.54740
**H**	-3.22906	-3.96137	-0.95602	**N**	-3.09145	3.62081	-0.07661
**H**	-3.00477	-3.64208	0.65262	**C**	0.51567	-0.18412	-0.13955
**H**	-2.67520	-1.66913	-1.56075	**N**	1.71833	-0.74525	0.47633
**H**	-1.34784	-2.46882	-0.69104	**C**	2.94180	-0.49146	-0.28254
**H**	-2.28268	-1.42577	1.48796	**C**	4.12731	-1.21170	0.35770
**H**	-3.40914	-0.51231	0.47471	**N**	5.36964	-0.93363	-0.39185
**H**	-0.96846	2.04044	0.59594	**H**	-4.48727	-3.62416	-0.75767
**H**	-2.35970	1.44973	1.48782	**H**	-3.26466	-1.57827	-1.58719
**H**	0.01983	-1.38631	0.77900	**H**	-2.39714	-2.62918	-0.46944
**H**	0.00619	0.05952	1.77568	**H**	-3.13641	-1.17279	1.44270
**H**	-3.61254	1.35642	-0.77009	**H**	-4.02140	-0.16584	0.30347
**H**	-2.17749	2.06770	-1.50363	**H**	-1.16847	1.90570	0.65922
**H**	-3.47381	3.91066	-0.97326	**H**	-2.67171	1.51753	1.48298
**H**	-3.83044	3.39085	0.53942	**H**	-0.81196	-1.62794	0.70264
**H**	0.96864	1.33236	-0.23934	**H**	-0.57468	-0.22624	1.73628
**H**	0.98763	-0.19163	-1.11794	**H**	-3.82885	1.73263	-0.81577
**H**	2.44434	0.26374	1.38201	**H**	-2.28001	2.27612	-1.45243
**H**	3.39668	-0.33211	-1.32064	**H**	-3.45198	4.21279	-0.81646
**H**	3.55621	1.26371	-0.58440	**H**	-3.75931	3.65371	0.68749
**H**	4.90689	0.29126	1.26135	**H**	0.58226	0.90800	-0.27716
**H**	4.74207	-1.31478	0.55042	**H**	0.40347	-0.61842	-1.13786
**H**	6.81506	0.06763	-0.02031	**H**	1.82296	-0.36254	1.41242
**H**	6.02336	-0.49147	-1.33515	**H**	2.79085	-0.88204	-1.29683
**H**	-4.24409	-2.92155	-0.16629	**H**	3.17827	0.57902	-0.38287
		PEI1v2$^+$		**H**	4.27141	-0.83893	1.37685
**N**	-3.56361	-3.13744	-0.22221	**H**	3.89721	-2.28200	0.42844
**C**	-2.49688	-2.19512	-0.56418	**H**	6.15545	-1.41053	0.04079
**C**	-2.53114	-1.00851	0.40207	**H**	5.29815	-1.29532	-1.33929
**N**	-1.48490	-0.01368	0.12803	**H**	-5.30754	-2.25090	-0.43514
**C**	-1.88710	1.35025	0.49605	**O**	5.62277	1.93772	-0.10192
**C**	-0.19107	-0.38441	0.69027	**H**	6.45649	2.28107	-0.43421
**C**	-2.76153	2.02573	-0.56250	**H**	5.64167	0.97418	-0.27986
**N**	-3.08279	3.39025	-0.13113			***H_2O···PEI2***	
**C**	0.96359	0.14566	-0.16336	**N**	-3.47556	3.15727	0.21838
**N**	2.25565	-0.14002	0.48752	**C**	-2.47780	2.13453	0.55832
**C**	3.41420	0.33792	-0.23554	**C**	-2.61483	0.93894	-0.38894
**C**	4.63146	-0.44559	0.25632	**N**	-1.64152	-0.12313	-0.11202
**N**	5.90416	0.03681	-0.44307	**C**	-2.11749	-1.44857	-0.51543

**H**	-3.70548	-3.82317	-0.95463	**C**	-0.31325	0.18459	-0.64536
**H**	-3.36060	-3.64237	0.63416	**C**	-3.03158	-2.09757	0.52604
**H**	-2.67950	-1.81942	-1.57429	**N**	-3.49528	-3.40155	0.03574
**H**	-1.49168	-2.64749	-0.57153	**C**	0.80655	-0.42122	0.20343
**H**	-2.47193	-1.37224	1.44404	**N**	2.12185	-0.12959	-0.38717
**H**	-3.50660	-0.53157	0.30152	**C**	3.23971	-0.69733	0.37832
**H**	-0.99487	1.96819	0.62153	**C**	4.58029	-0.39631	-0.28946
**H**	-2.40660	1.35961	1.47112	**N**	5.67480	-0.96549	0.50403
**H**	-0.11226	-1.47247	0.73160	**H**	-3.27860	4.03052	0.69431
**H**	-0.07975	-0.02317	1.73129	**H**	-2.54630	1.76699	1.59224
**H**	-3.64668	1.40743	-0.77052	**H**	-1.48928	2.58726	0.44734
**H**	-2.19189	2.08442	-1.49403	**H**	-2.55110	1.29542	-1.43096
**H**	-3.51046	3.91883	-0.88336	**H**	-3.61880	0.52085	-0.26651
**H**	-3.74411	3.38571	0.63936	**H**	-1.26095	-2.11042	-0.65826
**H**	0.82987	1.22054	-0.35149	**H**	-2.63898	-1.40556	-1.49053
**H**	0.94663	-0.35934	-1.13300	**H**	-0.17396	1.26696	-0.65784
**H**	2.24725	0.22924	1.43409	**H**	-0.21486	-0.15602	-1.69416
**H**	3.26096	0.13435	-1.30117	**H**	-3.85018	-1.40995	0.78616
**H**	3.59940	1.42475	-0.14019	**H**	-2.45344	-2.25919	1.43968
**H**	4.80341	-0.31057	1.32465	**H**	-3.98287	-3.91300	0.76276
**H**	4.53285	-1.50837	0.04061	**H**	-4.14277	-3.29143	-0.73862
**H**	6.73186	-0.47000	-0.11646	**H**	0.65114	-1.50304	0.33335
**H**	5.83932	-0.09519	-1.45718	**H**	0.77248	0.02851	1.19951
**H**	6.06212	1.03531	-0.27502	**H**	2.14657	-0.50409	-1.33335
	$PEI2^{+}$			**H**	3.22142	-0.24324	1.37552
**N**	-3.74962	-2.97438	-0.26694	**H**	3.14435	-1.78440	0.51732
**C**	-2.60720	-2.11618	-0.56594	**H**	4.60466	-0.87091	-1.27701
**C**	-2.58952	-0.93381	0.40690	**H**	4.66128	0.68875	-0.44842
**N**	-1.47233	-0.00371	0.16510	**H**	6.55511	-0.90727	0.00442
**C**	-1.78482	1.39034	0.52291	**H**	5.79213	-0.45510	1.37384
**C**	-0.21617	-0.45602	0.73398	**H**	-4.40755	2.86737	0.49854
**C**	-2.59571	2.12335	-0.54906	**O**	2.20220	2.73110	-0.07876
**N**	-2.78657	3.51459	-0.13458	**H**	2.48665	3.19268	-0.87241
**C**	0.93471	-0.02902	-0.18263	**H**	2.23664	1.77462	-0.30109
**N**	2.28390	-0.33108	0.44667		***H_2O···PEI3***		
**C**	3.48564	0.07930	-0.37988	**N**	-3.48547	3.07916	-0.41784
**C**	4.80418	-0.27316	0.31849	**C**	-2.36515	2.25876	0.06605
**N**	5.89147	0.15389	-0.54550	**C**	-2.35632	0.93117	-0.69814
**H**	-3.95587	-3.60993	-1.02817	**N**	-1.30635	-0.01248	-0.25973
**H**	-3.60828	-3.52858	0.57058	**C**	-1.63157	-1.38911	-0.68079
**H**	-2.72465	-1.72344	-1.57902	**C**	0.01757	0.39182	-0.77428
**H**	-1.63673	-2.64304	-0.54315	**C**	-2.63995	-2.09565	0.22853
**H**	-2.58337	-1.29953	1.44826	**N**	-2.92841	-3.42806	-0.31475
**H**	-3.52487	-0.38956	0.28021	**C**	1.18088	-0.13269	0.07076
**H**	-0.85369	1.94833	0.65654	**N**	2.45250	0.21170	-0.55995
**H**	-2.31070	1.43652	1.49128	**C**	3.62511	-0.17198	0.22388
**H**	-0.22546	-1.54735	0.80807	**C**	4.91551	0.26499	-0.46738
**H**	-0.04886	-0.06663	1.75734	**N**	6.08011	-0.13078	0.33354
**H**	-3.53280	1.58461	-0.74896	**H**	-3.41285	4.02798	-0.06740
**H**	-2.02367	2.11612	-1.48092	**H**	-2.39574	2.05543	1.14333
**H**	-3.10291	4.08805	-0.90827	**H**	-1.44494	2.81505	-0.13099

**H**	-3.48451	3.59331	0.59828	**H**	-2.27787	1.13471	-1.77794
**H**	0.91942	1.04321	-0.37599	**H**	-3.32513	0.44943	-0.54035
**H**	0.89718	-0.55878	-1.13388	**H**	-0.71824	-1.98558	-0.68368
**H**	2.32981	0.13394	1.35838	**H**	-2.00803	-1.39097	-1.71946
**H**	3.39395	-0.43093	-1.33961	**H**	0.07830	1.48019	-0.78434
**H**	3.41350	1.15331	-0.54747	**H**	0.13600	0.06333	-1.82178
**H**	4.87013	0.27628	1.26270	**H**	-3.53555	-1.47284	0.36404
**H**	4.81889	-1.34630	0.56876	**H**	-2.19615	-2.22320	1.21921
**H**	6.71339	0.43372	-0.02587	**H**	-3.47101	-3.97939	0.34122
**H**	6.16407	-0.55340	-1.21778	**H**	-3.47061	-3.36346	-1.17147
**H**	2.34133	-1.33392	0.64685	**H**	1.07475	-1.21809	0.24227
	$PEI3^+$			**H**	1.14428	0.34519	1.05392
**N**	-3.37674	-3.24887	-0.07944	**H**	2.50381	-0.22376	-1.47715
**C**	-2.34486	-2.31398	-0.50458	**H**	3.55300	0.32671	1.19823
**C**	-2.44833	-1.07453	0.39228	**H**	3.67893	-1.25451	0.42547
**N**	-1.42286	-0.00570	0.05166	**H**	4.99269	-0.24139	-1.43628
**C**	-1.89570	1.38184	0.45635	**H**	4.85539	1.34343	-0.67214
**C**	-0.05238	-0.32699	0.62779	**H**	6.94314	0.03502	-0.17224
**C**	-2.87764	1.99728	-0.54782	**H**	6.12934	0.41083	1.19094
**N**	-3.25368	3.30997	-0.04334	**H**	-4.37128	2.71043	-0.08488
**C**	1.08563	0.29694	-0.18262	**O**	-1.29973	0.27435	2.66296
**N**	2.34196	-0.03803	0.46174	**H**	-1.26835	0.10228	1.69705
**C**	3.52909	0.18845	-0.37774	**H**	-0.95918	-0.52235	3.07994
**C**	4.80974	-0.16628	0.37914		***$H_2O\cdots PEI1^+$***		
**N**	5.96851	0.07462	-0.47937	**N**	3.46177	-2.15781	0.98979
**H**	-3.70129	-3.83377	-0.83915	**C**	2.36528	-1.11161	1.11769
**H**	-3.06949	-3.85355	0.67383	**C**	2.30229	-0.22768	-0.13067
**H**	-2.55035	-2.01901	-1.53892	**N**	1.13660	0.63243	-0.04532
**H**	-1.32668	-2.72781	-0.48495	**C**	1.35631	1.97252	-0.61722
**H**	-2.29782	-1.32395	1.44404	**C**	-0.08498	-0.02123	-0.54341
**H**	-3.43906	-0.63795	0.28247	**C**	2.09124	2.91902	0.33616
**H**	-1.02101	2.02390	0.53856	**N**	2.26019	4.21945	-0.30554
**H**	-2.34269	1.27993	1.44670	**C**	-1.35523	0.45827	0.16158
**H**	0.06300	-1.40890	0.63823	**N**	-2.51442	-0.24337	-0.37799
**H**	-0.06288	0.02826	1.65987	**C**	-3.76274	0.04188	0.33802
**H**	-3.72787	1.32386	-0.72730	**C**	-4.93861	-0.71270	-0.28185
**H**	-2.36517	2.12852	-1.50655	**N**	-6.17183	-0.39954	0.44258
**H**	-3.47979	3.95752	-0.78784	**H**	3.57321	-2.67989	1.86154
**H**	-4.04220	3.27316	0.59241	**H**	3.24605	-2.84755	0.23164
**H**	0.92719	1.38070	-0.31983	**H**	2.57548	-0.51643	2.00552
**H**	1.09177	-0.14970	-1.18415	**H**	1.43681	-1.66020	1.27216
**H**	2.43933	0.48324	1.32893	**H**	2.30361	-0.85571	-1.04149
**H**	3.44052	-0.45325	-1.26167	**H**	3.20515	0.38873	-0.17181
**H**	3.60719	1.22543	-0.73498	**H**	0.38615	2.41638	-0.83859
**H**	4.89876	0.48829	1.25306	**H**	1.89498	1.91000	-1.57870
**H**	4.72810	-1.19573	0.75652	**H**	-0.00981	-1.10000	-0.37740
**H**	6.82389	0.14213	0.05918	**H**	-0.18161	0.11651	-1.63398
**H**	6.09500	-0.67054	-1.15606	**H**	3.03894	2.46195	0.67167
**H**	-1.32211	-0.00103	-0.96792	**H**	1.47538	3.05047	1.22929
	H_2O			**H**	2.48965	4.94494	0.36303
**O**	0.00000	0.00000	0.11708	**H**	2.98594	4.20953	-1.01388

**H**	0.00000	0.76350	-0.46830	**H**	-1.44164	1.55525	0.09209
**H**	0.00000	-0.76350	-0.46830	**H**	-1.27366	0.22205	1.22742
	PSS···PEI1⁺			**H**	-2.63392	0.00423	-1.35702
**C**	-4.41057	-1.36005	1.13024	**H**	-3.63394	-0.28022	1.37828
**C**	-5.26191	-0.29579	1.42233	**H**	-4.00704	1.11481	0.36409
**S**	-2.16946	-2.51287	0.01614	**H**	-5.06866	-0.38003	-1.31775
**O**	-1.62124	-2.28085	-1.37262	**H**	-4.69340	-1.78447	-0.31813
**O**	-1.02941	-2.39460	1.00873	**H**	-6.99101	-0.70000	-0.07279
**O**	-2.95535	-3.74183	0.17014	**H**	-6.19830	-0.86016	1.34636
**H**	-7.87074	2.32226	-1.67057	**H**	4.36258	-1.72264	0.77661
**H**	-6.82061	0.98211	-1.19713	**O**	2.83880	-4.04731	-0.95150
**H**	-8.23936	1.37985	-0.22359	**H**	2.20666	-3.95613	-1.67454
**H**	-7.21123	3.61511	0.35011	**H**	3.19443	-4.94171	-1.01804
**H**	-5.79413	3.21471	-0.59767			***H_2O···PEI1v2⁺***	
**H**	-6.76548	1.72039	1.87821	**N**	-4.54572	-2.77271	-0.11149
**H**	-4.82992	2.77647	3.05018	**C**	-3.40230	-1.95473	-0.52345
**H**	-5.96623	3.99051	2.43837	**C**	-3.24048	-0.77000	0.43343
**H**	-4.43937	3.69986	1.59569	**N**	-2.06078	0.05238	0.12914
**H**	-3.63518	2.17959	-0.22341	**C**	-2.23785	1.46453	0.48976
**H**	-2.12729	0.29964	-0.75517	**C**	-0.83259	-0.51438	0.67705
**H**	-4.62417	-2.36073	1.48376	**C**	-2.99910	2.26364	-0.56944
**H**	-6.14784	-0.47654	2.02303	**N**	-3.09157	3.66724	-0.15256
**N**	0.88377	-2.93848	-0.71616	**C**	0.38412	-0.17251	-0.18545
**C**	2.10263	-2.11236	-0.95681	**N**	1.61628	-0.67297	0.44707
**C**	1.77900	-0.62218	-0.82945	**C**	2.83054	-0.36533	-0.28115
**N**	2.98440	0.17768	-1.03743	**C**	3.94360	-1.27742	0.23293
**C**	2.70615	1.45743	-1.70133	**N**	5.25576	-0.98346	-0.47333
**C**	3.78042	0.32456	0.18786	**H**	-4.58382	-3.64321	-0.62936
**C**	2.57694	1.31905	-3.22004	**H**	-3.46738	-1.56548	-1.55028
**N**	2.24852	2.62000	-3.80941	**H**	-2.51133	-2.58889	-0.48059
**C**	5.27780	0.46657	-0.09145	**H**	-3.22451	-1.14316	1.47001
**N**	6.01330	0.62624	1.16004	**H**	-4.12756	-0.13648	0.34715
**C**	7.46383	0.69873	0.98934	**H**	-1.25832	1.93302	0.60632
**C**	8.16961	0.84069	2.33708	**H**	-2.74366	1.56355	1.46755
**N**	9.62246	0.90444	2.14287	**H**	-0.92417	-1.60146	0.72040
**H**	1.07194	-3.93722	-0.78478	**H**	-0.65731	-0.17640	1.71701
**H**	0.39032	-2.74986	0.20918	**H**	-3.97523	1.79565	-0.76539
**H**	2.47973	-2.32722	-1.95770	**H**	-2.43555	2.21903	-1.50565
**H**	2.86103	-2.41445	-0.23313	**H**	-3.44608	4.24824	-0.90391
**H**	1.30473	-0.42384	0.14522	**H**	-3.72710	3.77656	0.63120
**H**	1.03978	-0.36455	-1.59115	**H**	0.42662	0.91225	-0.36485
**H**	3.52578	2.14732	-1.49876	**H**	0.27427	-0.65724	-1.15968
**H**	1.79532	1.92928	-1.29163	**H**	1.67409	-0.31946	1.39801
**H**	3.64332	-0.56056	0.81312	**H**	2.65362	-0.57980	-1.34154
**H**	3.42646	1.18095	0.78935	**H**	3.14710	0.69115	-0.21557
**H**	1.84910	0.53002	-3.46534	**H**	4.12834	-1.13061	1.29824
**H**	3.54096	0.99289	-3.61863	**H**	3.69813	-2.32435	0.05968
**H**	2.33696	2.59795	-4.81912	**H**	5.98779	-1.63442	-0.18164
**H**	1.29247	2.88871	-3.59751	**H**	5.15188	-1.08065	-1.48608
**H**	5.45749	1.29284	-0.80009	**H**	-5.42368	-2.28968	-0.27408
**H**	5.62882	-0.44569	-0.58482	**O**	6.24284	1.56186	0.06931

**H**	5.69035	1.46318	1.63781	**H**	6.59803	1.88251	0.90712
**H**	7.79106	-0.23107	0.50787	**H**	6.33215	2.29136	-0.55615
**H**	7.78357	1.52401	0.33296	**H**	5.59740	-0.01301	-0.27626
**H**	7.85766	1.77945	2.80919		***H_2O···PEI2$^+$***		
**H**	7.83833	0.02471	2.99527	**N**	3.93514	3.07191	0.34935
**H**	10.09459	1.15699	3.00415	**C**	2.90583	2.22867	-0.25688
**H**	9.98771	0.00194	1.85631	**C**	2.79973	0.91080	0.51657
**H**	0.07256	-2.70820	-1.34890	**N**	1.69806	0.05525	0.04007
	PSS···PEI1v2$^+$			**C**	1.97064	-1.38371	0.17467
**C**	9.08985	0.27631	2.36914	**C**	0.41280	0.43015	0.60808
**C**	9.26471	1.20216	1.16242	**C**	2.83565	-1.94481	-0.95658
**C**	8.78157	0.59957	-0.17721	**N**	2.98159	-3.39134	-0.77711
**C**	9.13476	1.52890	-1.35358	**C**	-0.69462	0.18899	-0.42290
**C**	7.30019	0.24613	-0.16199	**N**	-2.06653	0.39199	0.18080
**C**	6.31319	1.23052	-0.00500	**C**	-3.21083	0.33184	-0.80520
**C**	4.96211	0.90189	0.02070	**C**	-4.56383	0.60569	-0.12093
**C**	4.58113	-0.43222	-0.11368	**N**	-5.70228	0.59477	-1.01064
**C**	5.53727	-1.43006	-0.27348	**H**	3.91636	4.01234	-0.02814
**C**	6.88641	-1.08320	-0.29786	**H**	3.06802	1.99760	-1.32010
**S**	2.84373	-0.89604	-0.07968	**H**	1.95670	2.77233	-0.19359
**O**	2.15429	0.01050	0.89197	**H**	2.71259	1.12787	1.59179
**O**	2.29138	-0.62433	-1.47526	**H**	3.73276	0.35708	0.38754
**O**	2.78008	-2.32888	0.26710	**H**	1.02586	-1.93405	0.15581
**H**	9.47517	0.74037	3.28133	**H**	2.43478	-1.60674	1.15071
**H**	8.03768	0.03055	2.53710	**H**	0.43090	1.49405	0.86281
**H**	9.62929	-0.66450	2.22195	**H**	0.18744	-0.11763	1.54095
**H**	10.32451	1.45976	1.05288	**H**	3.79153	-1.40367	-1.00624
**H**	8.73970	2.14720	1.34334	**H**	2.32313	-1.76426	-1.90533
**H**	9.32930	-0.34031	-0.32392	**H**	3.34358	-3.83020	-1.61621
**H**	8.81669	1.09990	-2.30723	**H**	3.62840	-3.60979	-0.02575
**H**	10.21481	1.69531	-1.39775	**H**	-0.67198	-0.83168	-0.80488
**H**	8.65133	2.50497	-1.24976	**H**	-0.59569	0.87522	-1.26372
**H**	6.60084	2.27083	0.10997	**H**	-2.21932	-0.32439	0.91945
**H**	4.20759	1.66711	0.16311	**H**	-3.00502	1.06927	-1.58330
**H**	5.22233	-2.46352	-0.35751	**H**	-3.18711	-0.66246	-1.25452
**H**	7.63100	-1.86564	-0.41588	**H**	-4.72708	-0.14062	0.66251
**N**	-9.37899	3.48136	0.44169	**H**	-4.52463	1.58313	0.37495
**C**	-8.30954	2.51055	0.70565	**H**	-5.68481	1.31677	-1.72028
**C**	-8.52006	1.26493	-0.15741	**H**	4.86564	2.70197	0.18402
**N**	-7.47725	0.25292	0.03412	**O**	-2.54173	-1.65196	2.09524
**C**	-7.97050	-1.11186	-0.17960	**H**	-2.33480	-2.58945	1.99830
**C**	-6.26021	0.53542	-0.72863	**H**	-2.80846	-1.53595	3.01473
**C**	-8.70246	-1.68102	1.03818	**H**	-5.89052	-0.30512	-1.43418
**N**	-9.18032	-3.03709	0.73918	**H**	-2.08850	1.30020	0.65250
**C**	-5.00488	0.02982	-0.01178		***H_2O···PEI3$^+$***		
**N**	-3.82152	0.18206	-0.86444	**N**	3.28550	-3.26846	-0.35215
**C**	-2.59151	-0.27558	-0.23710	**C**	2.25166	-2.37418	0.14961
**C**	-1.39967	-0.03076	-1.16154	**C**	2.32773	-1.08189	-0.67166
**N**	-0.14300	-0.57152	-0.57760	**N**	1.31520	-0.02994	-0.26358
**H**	-9.34883	4.24258	1.11262	**C**	1.72146	1.34854	-0.75127
**H**	-9.28709	3.88750	-0.48379	**C**	-0.07383	-0.38989	-0.75683

**H**	-8.36085	2.21434	1.75621	**C**	2.83084	2.00680	0.07744
**H**	-7.30043	2.91727	0.53925	**N**	3.05799	3.33406	-0.48250
**H**	-8.60291	1.55792	-1.22196	**C**	-1.19935	0.26599	0.04674
**H**	-9.48466	0.83762	0.11529	**N**	-2.46241	-0.09355	-0.57308
**H**	-7.12593	-1.77040	-0.39129	**C**	-3.65188	0.18723	0.24176
**H**	-8.62961	-1.16277	-1.06648	**C**	-4.93058	-0.23483	-0.49995
**H**	-6.16146	1.61452	-0.86808	**N**	-6.17823	-0.01465	0.21454
**H**	-6.31338	0.09580	-1.74330	**H**	3.09171	-4.23838	-0.13354
**H**	-9.50193	-0.99303	1.35090	**H**	2.33152	-2.13697	1.22213
**H**	-7.99432	-1.73923	1.86866	**H**	1.28483	-2.86119	0.00358
**H**	-9.51705	-3.50159	1.57817	**H**	2.16950	-1.29428	-1.72950
**H**	-9.95423	-3.01347	0.07996	**H**	3.31606	-0.63639	-0.55957
**H**	-5.15324	-1.01359	0.31122	**H**	0.84084	1.98647	-0.71780
**H**	-4.85667	0.62208	0.89519	**H**	2.01859	1.23340	-1.79607
**H**	-3.97542	-0.32425	-1.73663	**H**	-0.18379	-1.47071	-0.70222
**H**	-2.45196	0.28564	0.69265	**H**	-0.11212	-0.09914	-1.80880
**H**	-2.61952	-1.34534	0.03976	**H**	3.72970	1.37444	0.10637
**H**	-1.54808	-0.52503	-2.12506	**H**	2.48383	2.11695	1.10766
**H**	-1.25878	1.03350	-1.34581	**H**	3.43814	3.97508	0.20316
**H**	0.74866	-0.54602	-1.20516	**H**	3.68707	3.31870	-1.27723
**H**	0.20909	-0.07125	0.25731	**H**	-1.04275	1.35476	0.13849
**H**	-0.24398	-1.54964	-0.29704	**H**	-1.19316	-0.14363	1.06217
	PSS···PEI2^{+}			**H**	-2.55479	0.36273	-1.47638
**C**	7.07550	1.31748	-0.78229	**H**	-3.56808	-0.38637	1.17152
**C**	7.31368	-0.18170	-0.58247	**H**	-3.72660	1.25209	0.52245
**C**	6.56399	-0.78882	0.62329	**H**	-4.98742	0.30588	-1.45275
**C**	7.00267	-2.24556	0.86778	**H**	-4.85481	-1.29612	-0.75161
**C**	5.05051	-0.68815	0.49646	**H**	-6.23911	-0.55294	1.07077
**C**	4.36780	-1.28142	-0.57645	**H**	4.20639	-3.04248	0.00595
**C**	2.98291	-1.21917	-0.67379	**O**	1.22261	0.19896	2.66078
**C**	2.25481	-0.55518	0.31351	**H**	1.83932	-0.15316	3.31363
**C**	2.90386	0.04257	1.38702	**H**	0.51939	0.61724	3.17160
**C**	4.29537	-0.02680	1.46813	**H**	-6.34249	0.96179	0.43144
**S**	0.45620	-0.52905	0.21161	**H**	1.28482	0.00172	0.77065
**O**	0.13054	0.08592	-1.13560				
**O**	-0.01381	-1.91636	0.33236				
**O**	-0.00313	0.39160	1.30735				
**H**	7.64643	1.69388	-1.63553				
**H**	6.01945	1.53520	-0.96357				
**H**	7.38570	1.88652	0.10040				
**H**	8.38551	-0.36290	-0.44024				
**H**	7.03479	-0.72440	-1.49322				
**H**	6.84906	-0.20668	1.50849				
**H**	6.49225	-2.66904	1.73659				
**H**	8.08132	-2.29962	1.04416				
**H**	6.77297	-2.87931	0.00602				
**H**	4.92187	-1.80645	-1.34674				
**H**	2.46816	-1.68463	-1.50569				
**H**	2.32059	0.54684	2.14746				
**H**	4.80038	0.43750	2.30975				
**N**	-4.37161	-3.76903	-1.85588				

**C**	-4.38594	-2.30860	-1.71720
**C**	-4.67773	-1.93784	-0.26155
**N**	-4.62419	-0.48640	-0.02146
**C**	-5.48053	-0.07112	1.09629
**C**	-3.24556	-0.01951	0.13246
**C**	-6.95110	0.07759	0.69990
**N**	-7.73965	0.46532	1.87631
**C**	-3.09667	1.42101	-0.36723
**N**	-1.66933	1.86190	-0.29402
**C**	-1.42764	3.29373	-0.62725
**C**	0.06158	3.63714	-0.55055
**N**	0.23681	5.04161	-0.92852
**H**	-4.33281	-4.03855	-2.83335
**H**	-3.54921	-4.16279	-1.40713
**H**	-5.18746	-1.90898	-2.34523
**H**	-3.45164	-1.83126	-2.04583
**H**	-3.97716	-2.47259	0.40300
**H**	-5.67748	-2.30087	-0.01730
**H**	-5.14205	0.89775	1.47157
**H**	-5.39393	-0.77454	1.94518
**H**	-2.58644	-0.65744	-0.45669
**H**	-2.89259	-0.09657	1.17275
**H**	-7.30405	-0.84617	0.21932
**H**	-7.03272	0.87354	-0.04585
**H**	-8.69354	0.69118	1.61739
**H**	-7.78713	-0.29534	2.54678
**H**	-3.69324	2.11916	0.22405
**H**	-3.41764	1.49177	-1.40762
**H**	-1.26086	1.61717	0.63116
**H**	-1.81207	3.46559	-1.63621
**H**	-1.99708	3.91820	0.06307
**H**	0.40407	3.50985	0.47958
**H**	0.63247	2.92782	-1.16303
**H**	1.11579	5.40517	-0.58032
**H**	0.24404	5.15503	-1.93702
**H**	-1.06306	1.23378	-0.89603
	PSS···PEI3^{+}		
**N**	-1.77753	-3.58949	3.34528
**C**	-1.80233	-2.44219	2.43193
**C**	-2.65022	-2.83196	1.21676
**N**	-2.76172	-1.74945	0.17562
**C**	-3.10454	-2.33550	-1.17169
**C**	-3.74747	-0.69226	0.59789
**C**	-1.89911	-2.94039	-1.90263
**N**	-2.38532	-3.53098	-3.15327
**C**	-3.63960	0.61647	-0.18832
**N**	-4.58748	1.57153	0.38324
**C**	-4.37596	2.94911	-0.07667
**C**	-5.29929	3.91434	0.66387
**N**	-5.11879	5.27746	0.15083
**H**	-0.96332	-3.55670	3.94691

**H**	-2.59650	-3.61381	3.94297
**H**	-0.78727	-2.22721	2.09606
**H**	-2.18230	-1.52266	2.89574
**H**	-3.66774	-3.10490	1.51423
**H**	-2.19404	-3.70218	0.74810
**H**	-3.51627	-1.54285	-1.79266
**H**	-3.88970	-3.08118	-1.01277
**H**	-3.56584	-0.47176	1.64761
**H**	-4.74630	-1.13141	0.51088
**H**	-1.37527	-3.66096	-1.26095
**H**	-1.18936	-2.14318	-2.13277
**H**	-1.69969	-3.43697	-3.89210
**H**	-2.60522	-4.51575	-3.05591
**H**	-3.80199	0.45001	-1.26421
**H**	-2.63165	1.02616	-0.08361
**H**	-5.53801	1.29086	0.15580
**H**	-3.33182	3.20813	0.12759
**H**	-4.52763	3.06955	-1.16032
**H**	-6.34186	3.62875	0.48109
**H**	-5.12173	3.81140	1.74436
**H**	-5.77821	5.91729	0.57901
**H**	-4.18767	5.62251	0.36333
**H**	-1.78476	-1.28272	0.11378
**C**	6.83362	2.09326	-1.09312
**C**	7.08343	0.77817	-0.35021
**C**	6.14029	0.53750	0.84987
**C**	6.56892	-0.71247	1.64044
**C**	4.67581	0.46591	0.44130
**C**	4.21210	-0.52194	-0.43881
**C**	2.87393	-0.58217	-0.81527
**C**	1.97423	0.35190	-0.30591
**C**	2.40701	1.34390	0.56709
**C**	3.75133	1.39266	0.93348
**S**	0.23166	0.25903	-0.76032
**O**	0.17009	-0.29286	-2.13150
**O**	-0.36357	-0.73079	0.24036
**O**	-0.33307	1.61000	-0.57169
**H**	7.54287	2.21896	-1.91620
**H**	5.82499	2.13137	-1.51210
**H**	6.94747	2.95175	-0.42348
**H**	8.11515	0.76032	0.02012
**H**	6.99882	-0.06205	-1.04929
**H**	6.24178	1.40060	1.51921
**H**	5.92307	-0.87329	2.50808
**H**	7.59835	-0.60756	1.99613
**H**	6.52116	-1.61055	1.01815
**H**	4.90426	-1.25095	-0.84717
**H**	2.52649	-1.33399	-1.51334
**H**	1.69971	2.07657	0.93567
**H**	4.08880	2.17354	1.60791

Table S2. WBI and FBO values, and the interaction lengths (d).

		B3LYP/6-311++G**			
**Associate**		**Interaction**	**WBI**	**FBO**	**d, Å**
**H_2O**	**PEI1**	H///N	0.145	0.107	1.932
	**PEI1v2**	H///N	0.144	0.107	1.930
	**PEI2**	H///N	0.144	0.100	1.910
	**PEI3**	H///N	0.020	0.028	1.961
		O///H(CH_2)	0.006	0.001	2.585
	**$PEI1^+$**	O///H(NH_3^+)	0.160	0.122	1.734
	**$PEI1v2^+$**	O///H(NH_3^+)	0.159	0.121	1.737
	**$PEI2^+$**	O///H(NH_2^+)	0.128	0.102	1.802
	**$PEI3^+$**	O///H(NH^+)	0.094	0.081	1.901
		O///H(CH_2)	0.017	0.024	2.772
		O///H(CH_2)*	0.010	0.016	2.917
**PSS**	**H_2O**	O(SO_3^-)///H	0.068	0.054	2.081
		O*(SO_3^-)///H*	0.068	0.054	2.080
		H(ring)///O	0.015	0.017	2.932
	**$PEI1^+$**	O///H(NH_3^+)	0.201	0.135	1.668
		O*///H*(NH_3^+)	0.162	0.114	1.747
	**$PEI1v2^+$**	O///H(NH_3^+)	0.076	0.062	2.048
		O*///H*(NH_3^+)	0.277	0.172	1.568
	**$PEI2^+$**	O///H(NH_2^+)	0.192	0.130	1.673
		O*///H*(NH_2^+)	0.104	0.082	1.882
	**$PEI3^+$**	O///H(NH^+)	0.276	0.172	1.530
		O*///H(CH_2)	0.042	0.047	2.296
		O**///H(CH_2)*	0.035	0.035	2.421
